# Dynamic patterns of correlated activity in the prefrontal cortex encode information about social behavior

**DOI:** 10.1371/journal.pbio.3001235

**Published:** 2021-05-03

**Authors:** Nicholas A. Frost, Anna Haggart, Vikaas S. Sohal

**Affiliations:** 1 Department of Neurology, University of California, San Francisco, San Francisco, California, United States of America; 2 Center for Integrative Neuroscience, University of California, San Francisco, San Francisco, California, United States of America; 3 Weill Institute for Neurosciences, University of California, San Francisco, San Francisco, California, United States of America; 4 Kavli Institute for Fundamental Neuroscience, University of California, San Francisco, San Francisco, California, United States of America; 5 Department of Psychiatry, University of California, San Francisco, San Francisco, California, United States of America; Institute for Basic Science, REPUBLIC OF KOREA

## Abstract

New technologies make it possible to measure activity from many neurons simultaneously. One approach is to analyze simultaneously recorded neurons individually, then group together neurons which increase their activity during similar behaviors into an “ensemble.” However, this notion of an ensemble ignores the ability of neurons to act collectively and encode and transmit information in ways that are not reflected by their individual activity levels. We used microendoscopic GCaMP imaging to measure prefrontal activity while mice were either alone or engaged in social interaction. We developed an approach that combines a neural network classifier and surrogate (shuffled) datasets to characterize how neurons synergistically transmit information about social behavior. Notably, unlike optimal linear classifiers, a neural network classifier with a single linear hidden layer can discriminate network states which differ solely in patterns of coactivity, and not in the activity levels of individual neurons. Using this approach, we found that surrogate datasets which preserve behaviorally specific patterns of coactivity (correlations) outperform those which preserve behaviorally driven changes in activity levels but not correlated activity. Thus, social behavior elicits increases in correlated activity that are not explained simply by the activity levels of the underlying neurons, and prefrontal neurons act collectively to transmit information about socialization via these correlations. Notably, this ability of correlated activity to enhance the information transmitted by neuronal ensembles is diminished in mice lacking the autism-associated gene *Shank3*. These results show that synergy is an important concept for the coding of social behavior which can be disrupted in disease states, reveal a specific mechanism underlying this synergy (social behavior increases correlated activity within specific ensembles), and outline methods for studying how neurons within an ensemble can work together to encode information.

## Abstract 2

Behaviorally-specific patterns of correlated activity between prefrontal neurons normally enhance the information that neuronal ensembles transmit about social behavior. This study shows that in a mouse model of autism, individual neurons continue to encode social information, but this additional information carried by patterns of correlated activity is lost.

## Introduction

Activity in different neurons often exhibits precise temporal relationships that are modulated by behavior [1–3]. For example, subsets of neurons can exhibit correlated activity in which they become coactive at the same time or within short windows of time during specific behaviors. However, correlated activity can occur simply as a by-product of the interconnected nature of neuronal networks [4]. Thus, it is unclear whether correlated activity contributes in a meaningful way to information encoding [5] or whether it is simply a reflection of (and thus redundant with) changes in other variables, e.g., firing rates [6].

Many experimental and theoretical studies have studied how noise correlations, e.g., correlations between the trial-to-trial variability of different neurons, affect information coding [7–9]. Notably, the overall level of noise correlations can be modified by behavioral states such as attention [10]. From a population coding perspective, noise correlations can either impair or enhance decoding, depending on whether correlations are present between neurons that have similar or distinct tuning for a behavioral variable of interest. A different question, separate from that of whether the level of variability (noise) between neurons is correlated on a trial-by-trial basis, is whether specific subgroups of neurons exhibit correlations that vary dynamically over time based on a behavioral variable. For example, a group of neurons may exhibit correlated activity (coactivity that occurs more often than expected by chance) only during a specific behavior. Groups of coactive neurons represent an attractive computational unit for information processing because they should optimize temporal summation in downstream targets. Specifically, increases in coactivity can enhance temporal summation over and above any increases driven by increases in activity levels, and increases in correlated activity can enhance temporal summation even when overall activity levels remain unchanged. Thus, even if a group of neurons do not increase their activity levels during a behavioral state, they could increase their correlated activity, and thereby more effectively activate a common downstream target. In this manner, behaviorally driven changes in correlations can transmit information independent of changes in the activity levels of the underlying neurons. Whereas noise correlations, as their name suggests, reflect correlations in noise, i.e., variability that is unrelated to a behavioral variable of interest, behaviorally modulated correlations represent potentially important signals in neural networks.

While multiple studies have shown that behavior can modulate correlations [3,11], the functional significance of this has remained unclear, because changes in correlations might simply reflect (and be redundant with) variation in activity levels [6] rather than contributing additional information. One study demonstrated that pairs of cells representing overlapping spatial locations were coactivated more frequently when the location was novel than for familiar locations [12]. However, this study did not evaluate whether this correlated activity transmitted additional information beyond that conveyed by the activity levels of the constituent neurons themselves. Even with the advent of new technologies for simultaneously recording from large numbers of neurons in behaving animals, many studies still focus on information that is transmitted by changes in the activity levels of neurons, while ignoring contributions from correlated activity or other ways in which neurons can act collectively. In particular, it is now commonplace to identify “neuronal ensembles” by grouping together neurons which significantly increase or decrease their activity during the same behaviors or in response to the same stimuli. However, this approach ignores information that is transmitted collectively, e.g., it would not be sensitive to the hypothetical group of neurons described above, which increase their correlations during a specific behavior while keeping their activity levels constant. Thus, current methods for identifying neuronal ensembles might falsely conclude that a group of neurons do not encode a behavioral variable (when they do encode it collectively), incorrectly estimate the amount of information that is being encoded, and/or miss important mechanisms that contribute to encoding.

Correlations have been shown to contribute additional information for small groups (3 to 8 neurons) of cortical neurons [13]. Recently, a few studies have examined how correlations contribute to encoding within larger cortical ensembles, which have greater potential for both synergy and redundancy. One found that the identity of a conditioned stimulus was encoded in mean activity levels, but not in moment-to-moment patterns of coactivity [14]. In hippocampal region CA1, disrupting correlations impairs the decoding of position, head direction, and speed [15]. And during a 2-choice discrimination task with probabilistic outcomes, correlations between neurons in secondary motor region M2 enhance the encoding of the choice [16]. Thus, some studies have found evidence that correlations between neurons can augment the information those neurons encode about behavioral variables [13,15,16]. However, many of these hypothesize that noise correlations help disambiguate trial-by-trial variation in activity levels that is related to behavioral variables of interest, from variation that is attributable to “noise,” e.g., changes in other variables such as internal states. They did not specifically examine whether correlations might themselves act as signals, encoding unique information by increasing or decreasing in relation to specific behavioral variables, creating behaviorally specific patterns of coactivity. Thus, specific mechanisms through which cortical neurons can collectively encode information currently remain poorly described. A further challenge is the lack of methods for identifying neurons that contribute to the collective encoding of behavioral variables, but do so without significantly increasing or decreasing their activity levels. This makes it difficult to directly examine these neurons in order to assess whether they contribute to ensemble encoding by altering their correlations, generating behaviorally specific patterns of coactivity, etc.

The goal of this study is to address these 2 challenges related to neural encoding in the cortex by focusing on a subject that has already been well-studied: the encoding of rodent socialization by neurons in the medial prefrontal cortex (mPFC) [17–20]. Many studies have already shown that prefrontal neurons can significantly increase or decrease their activity levels during periods of social interaction [2,19,20] and/or in response to social stimuli such as odors [21]. However, these studies have not assessed whether prefrontal neurons might act collectively to encode additional information (beyond what activity levels encode), examined whether this might occur via behaviorally driven changes in correlated activity, or identified ensembles based on neurons’ ability to encode information collectively, rather than through increases or decreases in their individual activity levels.

The goal of this study is to examine a neuronal population that is already known to encode social behavior and determine whether this encoding occurs solely via changes in the activity levels of neurons versus whether additional information is transmitted collectively, and if so, how this occurs. These are critical questions, because if we do not understand the mechanisms which normally encode information, we cannot study how they are disrupted in disease states. Thus, by design, we focused on these foundational questions about the nature of information transmission, rather than more nuanced issues, e.g., does this encoding differ between subpopulations of prefrontal neurons, etc.

We used microendoscopic calcium imaging in freely moving mice to measure activity in prefrontal ensembles when mice were either alone or engaged in social behavior. We used a neural network classifier to quantify how well prefrontal ensembles would transmit information about behavior to downstream neurons. By examining the operation of this neural network and using surrogate datasets which preserve activity levels but either preserve or disrupt patterns of correlated activity, we find that behaviorally driven changes in correlations enhance the information transmitted by neuronal ensembles. Notably, this was disrupted in a mouse model of autism (*Shank3* knockout (KO) mice), even though large numbers of prefrontal neurons are still recruited by socialization in *Shank3* KO mice and encode socialization through changes in their activity levels. This illustrates that the ability of prefrontal neurons to collectively encode information may be selectively disrupted in pathological states.

## Results

### Social interaction recruits prefrontal ensembles

We implanted microendoscopes (nVoke; Inscopix, Palo Alto, CA) into the mPFC of adult wild-type (WT) C57/B6J mice to image calcium transients using GCaMP6f expressed under control of the human synapsin promotor. We imaged freely moving mice during an assay which sequentially introduced 2 novel juvenile mice to the home cage of the subject mouse, first during an initial (novel) epoch and then again during a subsequent (familiar) epoch. These 4 epochs of social interaction were interleaved with epochs during which the subject mouse was alone in its home cage (“home cage” epochs). The first 5 minutes of each interaction epoch was scored by a blinded observer, and each WT mouse spent approximately 10 minutes interacting with the juvenile mice (393 +/− 25 seconds during the novel epochs and 235 +/− 18 seconds during the familiar epochs, *p* = 0.00017, paired *t* test, *n* = 10 WT mice).

**Fig 1:**
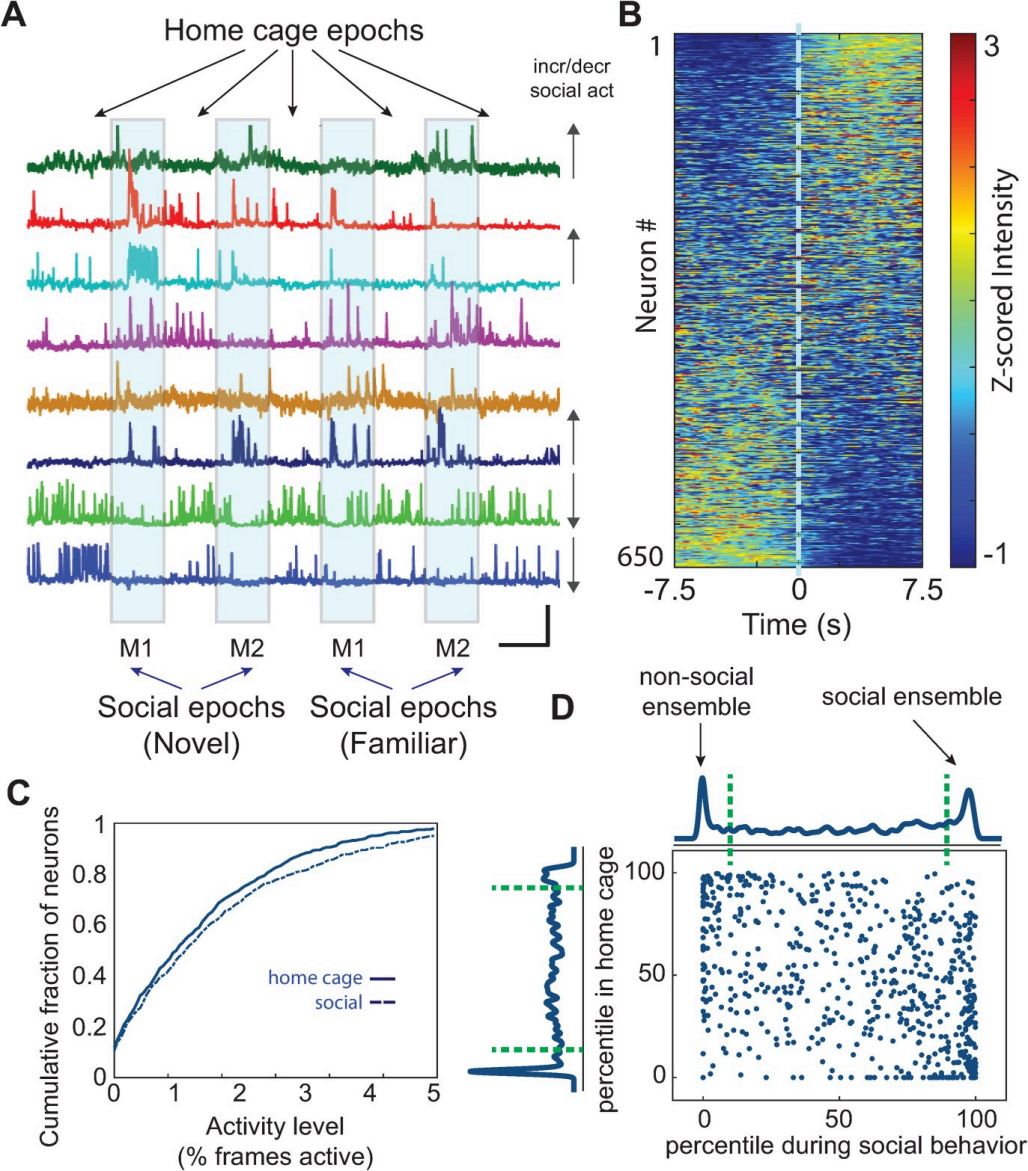
Social interaction modulates activity levels within prefrontal ensembles. **(A)** Mice were imaged across 9 consecutive behavioral epochs (each lasting 5 minutes) during which they were either alone in their HC or interacted with one of 2 novel sex-matched juvenile mice introduced to the HC (“M1” or “M2”). Each novel mouse was subsequently reintroduced to the HC during a familiar epoch. GCaMP traces show examples of neurons that appear to increase or decrease activity during social epochs (see arrows at the right of each trace). Scale bar: x-axis represents 5 minutes, and y-axis denotes maximum dF/F_0_ for each trace (scaled independently). **(B)** Mean z-scored GCaMP traces for all neurons recorded from WT mice (663 neurons from 10 mice) aligned to the onset of social interaction during the first bout of interaction within each social epoch. **(C)** Cumulative plot showing the distribution of activity levels for individual neurons during HC epochs or periods of social interaction (% time active in HC: 1.8 +/− 0.1%, % time active during social interaction: 2.1 +/− 0.1%, *p* = 0.00002, paired *t* test; *n* = 663 neurons from 10 WT mice). **(D)** Scatter plot showing the activity of each neuron during each behavioral condition, expressed as a percentile relative to a null distribution generated by circularly shuffling that neuron’s activity. Activity levels during social interaction or while the mouse was alone in its HC are plotted on the x-axis and y-axis, respectively. Kernel density plots along the axes indicate the fraction of neurons whose activity was at a given percentile of the null distribution. Neurons with activity >90th percentile of shuffled datasets (green dotted line) were considered to be positively modulated, whereas neurons with activity <10th percentile (green dotted line) were considered to be negatively modulated during each behavior (>90th percentile, social: 152/663 neurons, HC: 80/663 neurons; *p* < 0.00001, chi-squared test; <10th percentile, social: 128/663 neurons, HC: 119/663 neurons; *p* = 0.5, chi-squared test). Data used to generate this figure can be found in the Supporting information Excel spreadsheet (S1 Data). HC, home cage; WT, wild-type.

We processed data using a modified principal component analysis (PCA)/independent component analysis (ICA) approach [22,23] to identify neurons which were active during the imaging session. To minimize the influence of the surrounding neuropil on neuronal signals, we calculated the mean signal within each region of interest (ROI), then subtracted the mean signal calculated from a circular annulus surrounding each ROI (**S1 Fig**). Casual inspection of calcium traces revealed that some neurons were more active during epochs of social interaction (compared to periods of home cage exploration), whereas others exhibited the opposite pattern (**Fig 1A**). Correspondingly, aligning calcium traces to the onset of social interaction revealed many neurons that either increased or decreased their activity levels at the onset of interaction (**Fig 1B**). Fluorescence traces were converted to binary event rasters (see Materials and methods section for details of event detection), in which most neurons were “active” in less than 5% of frames (**Fig 1C**). As a population, imaged neurons were more active during social interaction (**Fig 1C**, *n* = 663 neurons from 10 mice, percent time active in home cage: 1.8 +/− 0.1%, percent time active during social interaction: 2.1 +/− 0.1%, *p* = 0.00002, paired *t* test). There was a bimodal distribution of cells that were significantly more (>90th percentile, social: 152/663 neurons, home cage: 80/663 neurons; *p* < 0.00001, chi-squared test) or less active (<10th percentile, social: 128/663 neurons, home cage: 119/663 neurons; *p* = 0.5) during either social interaction or matched periods when mice were alone in their home cage, as compared to circularly shuffled datasets (**Fig 1D**). These correspond to conventionally defined neuronal ensembles, i.e., groups of neurons that increased or decreased their activity levels during social interaction.

### Using a neural network classifier to assess how well ensembles transmit information

Next, we sought to determine how well these prefrontal ensembles would transmit information about social behavior to downstream neurons. Specifically, we measured how well downstream neurons could decode whether a mouse was engaged in social behavior based on input from these prefrontal neurons. Later, we will study how this was altered in *Shank3* KO mice. For this, we used a simple neural network classifier that received input from the recorded neurons. Our rationale for using this kind of neural network classifier was 3-fold. First, a simple neural network measures information that is immediately and readily available to downstream neurons. Second, for a neural network with only 1 hidden layer, it is straightforward to examine the weights to determine how the network performs the classification. This can provide insight into exactly how the neural network is able to decode behavior from the input activity. Third, examining how various parameters of the neural network affect its performance can provide additional clues about how information is represented within the input population.

**Fig 2:**
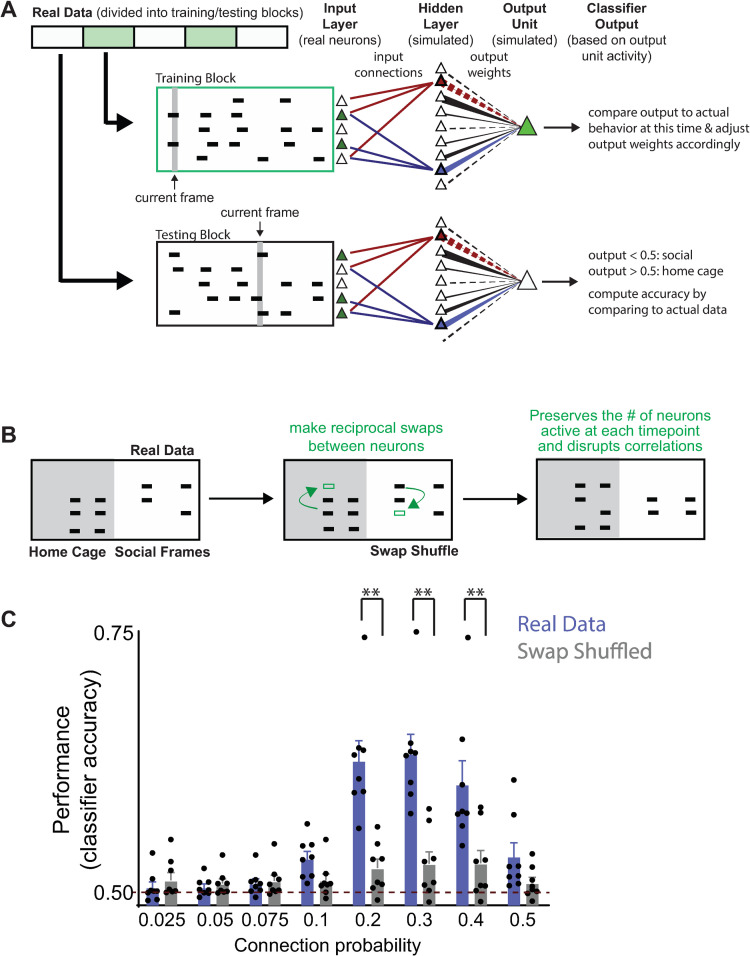
Classifying behavior from prefrontal ensembles using a simple neural network. **(A)** We constructed a neural network consisting of a single hidden layer (containing 1,000 units) connected to a single output unit. The thickness of lines between each hidden layer unit and the output unit reflects the magnitude of the output weight. Positive and negative weights are indicated by solid and dashed lines, respectively. Each hidden layer unit received input from a random subset of prefrontal neurons from one real dataset. For clarity, we have only shown input connections to 2 hidden layer units (which are differentiated by their blue and red colors)—output weights from other hidden units are shown in black. The output weight from each hidden layer unit was iteratively updated during training. We trained the classifier to distinguish periods marked as HC exploration or social interaction by dividing a dataset into 500-frame blocks and then using alternating blocks for training or testing. **(B)** Cartoon describing swap shuffling procedure. Note that in this figure, we swapped events between neurons and across the entire dataset, thereby disrupting behavior-specific changes in the activity levels of individual neurons, while preserving both the total activity for each neuron (over the entire experiment) and the number of neurons active in any given frame. **(C)** The classifier performed poorly (near chance) when the input connection probability (governing the number of prefrontal neurons that provided input to each hidden layer unit) was <10%. Classification accuracy was above chance in 8/10 mice and increased to a peak of 69 +/− 3% in these mice, before decreasing again for connection probabilities >30%. The classifier performance was significantly decreased to near-chance levels when we trained and tested using data that had been randomly swap shuffled (2 way RM ANOVA shuffle vs. real *p* < 0.0005; connection *p* < 0.0001, interaction *p* < 0.0001; follow-up with Bonferonni multiple comparisons test revealed significant differences for input connection probabilities of 20%, 30%, and 40% at *p* < 0.01). Data used to generate this figure can be found in the Supporting information Excel spreadsheet (S1 Data). HC, home cage; RM ANOVA, repeated measures analysis of variance.

**Fig 2A** shows the design of the neural network classifier. For clarity, we use the terms “neurons” specifically to refer to actual prefrontal neurons (which provide input to the neural network) and “units” to refer to simulated elements within the network. The network consisted of a hidden layer containing 1,000 units. We chose this number because it is both an order of magnitude larger than the number of input neurons and an order of magnitude smaller than the number of frames available for training (the latter helps ensure that there will be enough data to train the output weights). We simulated a different neural network for each mouse. Each hidden layer unit received input from a random subset of the prefrontal neurons from one mouse, i.e., each frame represents one time point, and if neuron *i* is active in a frame, then it provided an input of 1 to all the hidden units to which it is connected; otherwise, it provides an input of 0. For each simulation, there was a fixed connection probability between each input neuron and each hidden layer unit. We tried different values for this connection probability in order to measure how classifier performance depends on the number of neurons that provide input to each hidden layer unit. Each hidden layer unit had an output weight which specifies how strongly that unit excites or inhibits a single output unit which classifies activity as belonging to periods in which a mouse was actively engaged in social interaction or alone in its home cage. For example, output unit activity <0.5 corresponds to the social condition, while output unit activity >0.5 corresponds to the home cage condition. These output weights were adjusted during training via a gradient descent learning rule (Materials and methods), while the pattern of input connectivity was fixed. This models the situation in which prefrontal neurons transmit information to a downstream population of neurons (the hidden layer) that decode behavior via their output weights. We initially trained networks on 50% of the data (frames) and used the held-out data for testing. We trained and tested using intervals during which the mouse was actively engaged in social interaction (i.e., actively sniffing/nose in contact with subject mouse) or matched intervals when the mouse was alone in its home cage.

Because each hidden unit simply sums the activity of a fixed population of mPFC neurons, one can think of each hidden unit as representing one ensemble of mPFC neurons. The size of these ensembles is determined by the input connection probability. In this framework, training serves to determine how well each ensemble (corresponding to an individual hidden unit) is correlated with behavior. Hidden units corresponding to ensembles which are strongly associated with one type of behavior will be assigned large positive or negative output weights, whereas those that are not well correlated with behavior will have output weights near 0.

Critically, we did not attempt to classify more nuanced behaviors, besides whether the subject mouse was exploring another mouse versus alone in its home cage. This was by design because doing so would have reduced the number of training frames available for each behavioral label. A large number of training frames for each behavioral label are necessary to train a neural network with a large number of hidden units, and a large number of hidden units are necessary in order to effectively capture possible collective encoding (because, as noted above, each hidden unit effectively represents one neuronal ensemble). Thus, subdividing periods of social interaction into more nuanced behavior would have compromised our ability to resolve collective encoding. Therefore, because the focus of this paper is on trying to study collective encoding, we intentionally used simple, binary classifications of behavior (e.g., social versus home cage). As a control, we also trained and tested our classifier on shuffled datasets, using a method that preserved the activity of each neuron over the course of the experiment and the overall activity of the network (number of active neurons) in each frame (swap shuffle; **Fig 2B**).

### Classifier performance is optimal for intermediate connection probabilities

Classifier performance was strongly dependent on the probability that each input neuron was connected to each hidden unit. For the 8/10 datasets that could be classified above chance, classifier performance (measured on the 50% of data which was held out during training) was near chance levels when the connection probability was <0.1, but increased to a peak of 69 +/− 3% (**Fig 2C**) for a connection probability of 0.3. Accuracy decreased dramatically when the connection probability increased to 0.5 indicating that connection probabilities approximately 0.2 to 0.4 are optimal.

We also validated classifier performance by training and testing on surrogate datasets that were generated by “swap shuffling” our original datasets. We created “swap-shuffled” surrogate datasets by randomly swapping blocks of activity between neurons (each block of activity = a set of consecutive frames during which the neuron was active). To understand this, think of the entire raster as a collection of blocks of activity. Each block occurs at a specific time, has a specific duration, and is associated with a particular neuron. Swap shuffling is equivalent to just shuffling the neurons associated with each block of activity (the start time and duration of each block do not change). For example, if neuron *i* originally became active at time *t1* for *n1* frames and neuron *j* was active at time *t2* for *n2* frames, then in the surrogate dataset, neuron *i* might become active at *t2* (but not at *t1*), while neuron *j* might become active at *t1* (but not *t2*). Swap shuffling preserves the number of neurons active at each point in time (because the timing of blocks of activity does not change). It also preserves the number of blocks of activity for each neuron, and this tends to preserve the overall level of activity of each neuron. Activity levels are not perfectly preserved, because blocks of activity can have different durations. Nevertheless, in practice, blocks of activity tend to have similar durations and the similarity between the mean activity level in each neuron before and after swap shuffling of entire datasets was 0.97 +/− 0.01. Because in this case we have shuffled across frames from the entire experiment (all 9 epochs), this preserves the number of neurons active in every frame, but abolishes relationships between the activity of individual neurons and the underlying behavior. Residual above-chance classifier performance on datasets that have been swap shuffled reflects differences in the total level of network activity during distinct behaviors. For example, if there is more activity during the social interaction, the classifier can exploit this to correctly classify some frames, even when the identities of the particular neurons that are active at specific times have been shuffled. As expected, we found that neural network classifiers trained and tested on swap-shuffled datasets performed near, but slightly above, chance levels (**Fig 2B**; average 53.7 +/− 1.6% accuracy for input connection probability = 0.3, *p* = 0.06, and 53.1 +/− 1.2% for connection probability = 0.2, *p* = 0.04, *t* test).

### Prefrontal neurons that drive classifier performance change their correlations during social behavior

**Fig 3:**
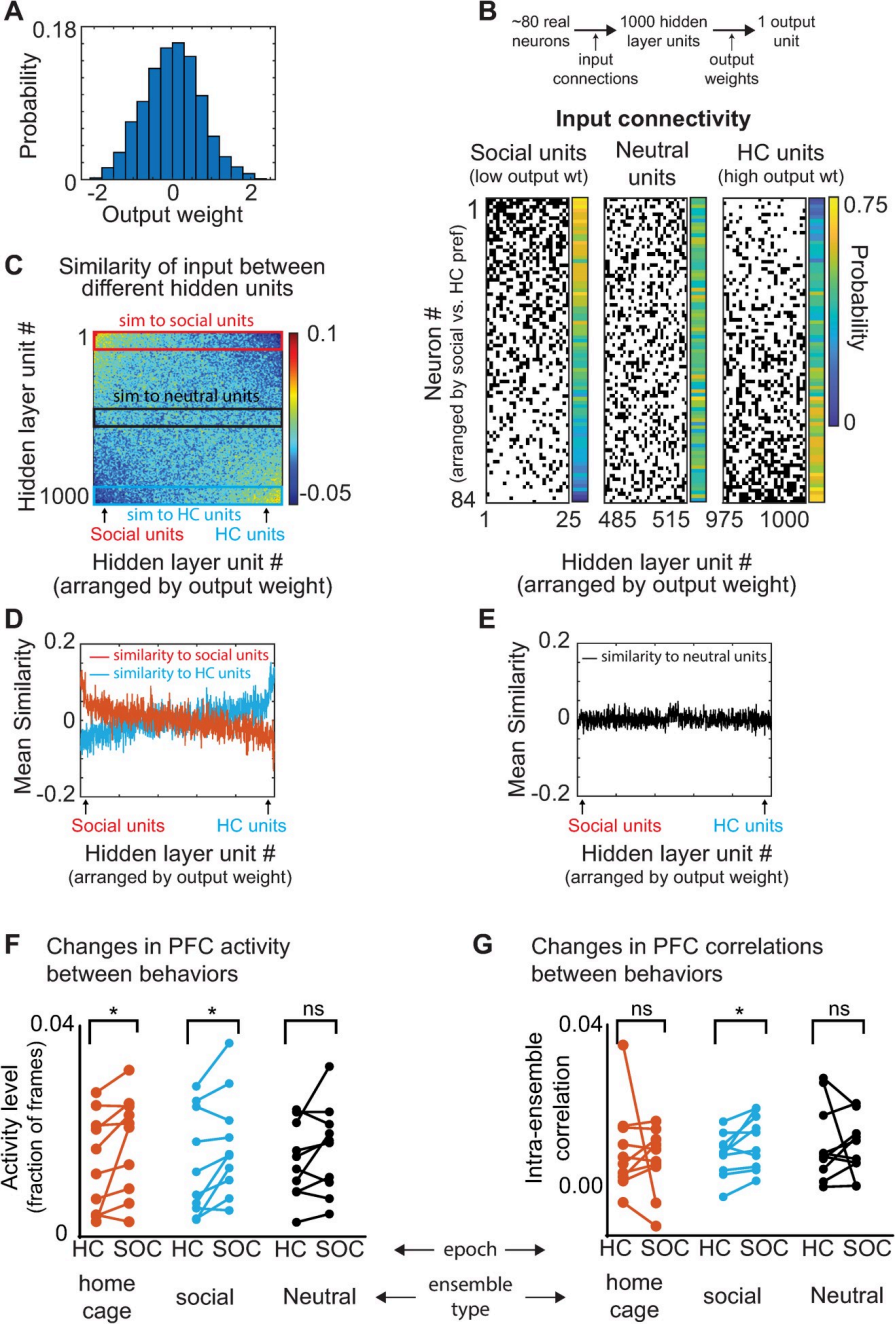
Classifier weights reveals an ensemble that increases correlations during social behavior and detects social behavior. **(A)** Example histogram depicting the distribution of output weights assigned to connections between hidden layer units and the output unit over the course of training. **(B)** Matrix of input connections for hidden units which detect the social (left) or HC condition (right). The hidden layer units (x-axis) have been arranged in order of increasing output weights to identify “social units” (25 most negative output weights) and “HC units” (25 largest positive output weights). We also plotted “neutral units” (30 units closest to 0). To the right of each matrix is the probability that a given neuron will be connected to a given set of units. Prefrontal neurons (y-axis) have been arranged in order of their preference for social interaction vs. HC, i.e., the difference in their probability of being connected to home cage versus social units. **(C)** Correlation matrix showing the input similarity, i.e., the pairwise correlation between binary vectors representing the input connections to each pair of hidden layer units. Hidden layer units are arranged in order of increasing output weight. Red and blue rectangles indicate the input similarity compared to social or HC units, respectively. A Gaussian filter with a standard deviation of 3 was applied to the 1,000 × 1,000 matrix to improve visualization. **(D)** For each hidden layer unit, we plotted its average input similarity to either the 25 social units (red) or the 25 HC units (blue). Hidden layer units (x-axis) are again arranged by output weight. Social units had similar patterns of input compared to each other but not to HC units and vice versa. **(E)** The average input similarity of each hidden layer unit to 25 hidden layer units with near-zero output weights (“neutral units”; black rectangle in C). **(F)** We defined social and HC ensembles as the 20% of prefrontal neurons most likely to provide input to the social or HC units, respectively. The mean activity of both HC and social ensembles increased during social interaction compared to the HC condition (social ensemble: mean activity level 1.4 +/− 0.3% in HC vs. 1.8 +/− 0.3% during social interaction, *p* < 0.05, sign-rank test; HC ensemble: mean activity level 1.5 +/− 0.30% in HC vs. 1.9 +/− 0.3% during interaction, *p* < 0.001, sign-rank test; neutral ensemble 1.5 +/− 0.2 in HC vs. 1.7+/− 0.3 during interaction, *p* = 0.16). **(G)** Correlations between neurons in the same ensemble increased during social interaction for the social ensemble (mean correlation between neurons in the social ensemble: 0.008 +/− 0.002 in HC vs. 0.012 +/− 0.002 during social interaction, *p* < 0.05; HC ensemble mean correlation 0.010 +/− 0.003 in HC vs. 0.007 +/− 0.003 during social interaction, *p* = 0.77, sign-rank test; neutral ensemble mean correlation coefficient 0.011 +/− 0.003 in HC vs. 0.010 +/− 0.002 during social interaction; *p* = 0.7). Note: A–E show a representative example from a single mouse. F and G represent data from a single iteration of the classifier at the optimal input connection probability (0.3) averaged over all mice. Data used to generate this figure can be found in the Supporting information Excel spreadsheets (S1 Data for panels F and G and S2 Data for panels C–E). HC, home cage; PFC, prefrontal cortex.

Next, we examined connections in trained networks to reveal factors which enable them to successfully classify social versus home cage behavior (we analyzed networks with input connection probability = 0.3 since this hyperparameter value optimized classifier performance). Each hidden layer unit has an output weight which measures how strongly it excites or inhibits the output unit that represents the “decision” (social versus home cage). Hidden units with output weights approximately equal to 0 do not contribute to this decision. By contrast, hidden units with strong negative or positive weights promote the social or home cage decision, respectively (**Fig 3A**). Therefore, we hypothesized that there might be important differences in the pattern of input to hidden units, depending on whether those hidden units have large positive or negative output weights.

We arranged hidden layer units based on their output weights, i.e., the unit with the most negative weight was unit 1 and the unit with the most positive weight was unit 1,000. Then, we defined the 25 hidden layer units with the most negative weights as “social units” and the 25 with the most positive weights as “home cage units” (**Fig 3B**). For comparison, we also defined the 25 hidden layer units with the weights closest to 0 as “neutral units.” For each pair of hidden units, we computed the similarity between their inputs (i.e., the correlation between their input vectors; **Fig 3C**). We then plotted the average input similarity of each hidden unit to either the social or home cage units (**Fig 3D**) or the neutral units (**Fig 3E**). Social and home cage units tended to receive input from the same prefrontal neurons as other hidden layer units with the same preference, i.e., which also had negative or positive output weights. By contrast, neutral units did not exhibit any such relationship.

The preceding suggests that distinct ensembles of prefrontal neurons provide input to either social or home cage hidden units. We hypothesized that there might be important features of activity in these ensembles that support the classification of social versus home cage behavior. For example, one possibility is that prefrontal neurons which provide input to social units might tend to increase activity during social behavior, whereas prefrontal neurons which provide input to home cage units do the opposite. Surprisingly, this was not the case. In fact, both ensembles of prefrontal neurons significantly increased their activity when mice were engaged in social interaction (**Fig 3F**; social ensemble: mean activity level 1.4 +/− 0.3% in home cage versus 1.8 +/− 0.3% during social interaction, *p* < 0.05, sign-rank test; home cage ensemble: mean activity level 1.5 +/− 0.3% in home cage versus 1.9 +/− 0.3% during social interaction, *p* < 0.001, sign-rank test). Next, we examined pairwise correlations between the activity of prefrontal neurons within each ensemble. Strikingly, mean correlations within the social ensemble increased during social interaction (**Fig 3G**; mean correlation coefficient between neurons in the social ensemble: 0.008 +/− 0.002 in home cage versus 0.012 +/− 0.002 during social interaction, *p* < 0.05). By contrast, there was a nonsignificant decrease in correlations within the home cage ensemble (**Fig 3G**; home cage ensemble mean correlation coefficient 0.010 +/− 0.003 in home cage versus 0.007 +/− 0.002 during social interaction, *p* = 0.77, sign-rank test).

Thus, the ensemble of prefrontal neurons which provide input to the social units form an assembly that collectively becomes more coactive (correlated) during social behavior. In contrast, the prefrontal neurons which provide input to the home cage units increase their activity, but not their coactivity, during social behavior. This suggests that behaviorally driven changes in correlations may contribute to the encoding of social behavior.

### Swap shuffling within a behavioral state preserves information encoded by activity levels but disrupts encoding by correlated activity

How can we quantitatively assess the contribution of these behaviorally modulated correlations to classifier performance? Ideally, we would first train a neural network on the original data. Then, we would test this network’s ability to classify data which maintained behaviorally driven changes in activity levels, but either removed or preserved the specific patterns of correlated activity found in the original dataset. Indeed, we have already developed methods for shuffling that achieve these goals. First, to shuffle the data in a manner that maintains behaviorally driven changes in activity levels, but disrupts the patterns of correlated activity, we can swap shuffle activity, but do so within each behavioral condition rather than across the entire testing dataset. In other words, we first divide up the raster into separate subrasters for each 5-minute behavior epoch (when the mouse was either engaged in social interaction or alone in its home cage). Then, we performed swap shuffling (as described above) separately on each subraster, before recombining these swap-shuffled subrasters to create the swap-shuffled surrogate dataset for testing. Because swap shuffling tends to preserve activity levels, and because we swap shuffled activity within a behavioral condition, neurons that increase or decrease activity during periods of social interaction in the original dataset will also do so in the swap-shuffled surrogate dataset.

**Fig 4:**
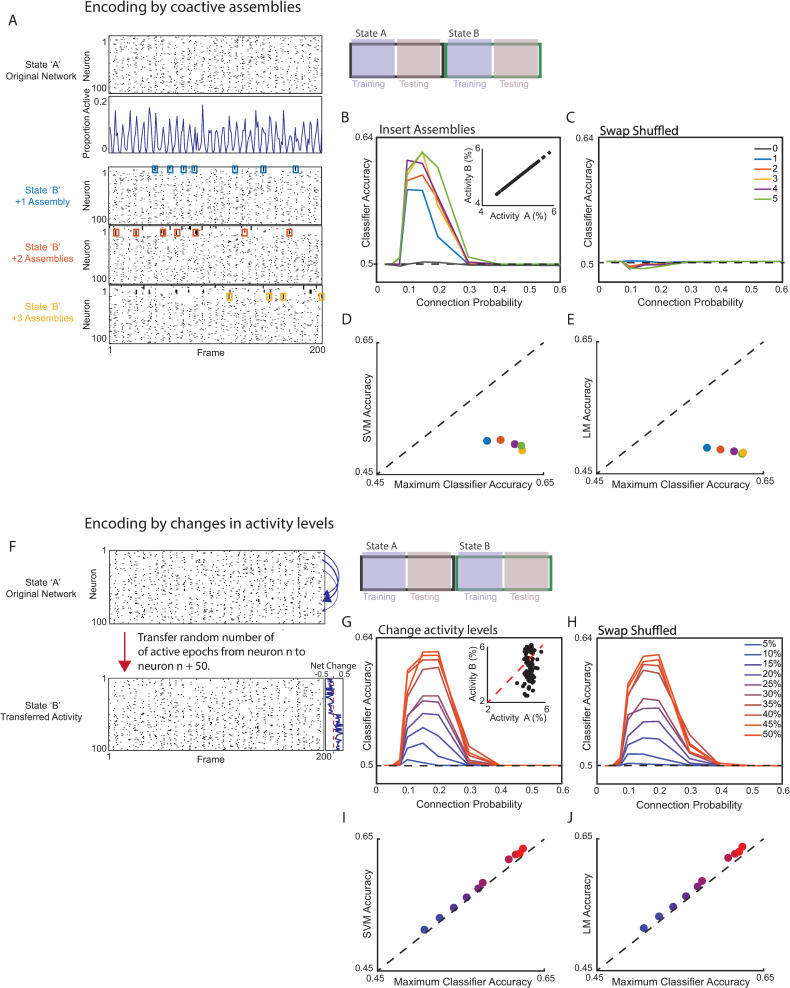
Neural network classifiers can distinguish states which differ in either activity or correlations. **(A)** We generated synthetic datasets consisting of 100 neurons. Each dataset consisted of 2 activity rasters, corresponding to States “A” and “B.” Each raster contained 6,000 time points (frames). The overall level of activity in each raster oscillated around a mean level (5% of neurons active in a frame). In “State A,” neuronal activity was randomly assigned. Then, we created 1–5 cell assemblies in “State B” by rearranging the activity raster for “State A.” Specifically, whenever the first neuron in an assembly was active, we swapped activity across the rest of the network so that other neurons in the same assembly would be coactive. This was achieved by making reciprocal swaps between neurons. For example, suppose a neuron outside the assembly was active in the desired frame. Then, we swapped its activity with a neuron within the assembly that was active in a different frame. Thus, neither the total number of neurons active in a given frame nor the total activity level of any neuron differed between the “State A” and “State B” rasters. As a result, “State B” was differentiated from “State A” only by the presence of 1, 2, 3, 4, or 5 assemblies comprised of correlated (coactive) neurons. **(B)** A neural network classifier performed above chance in distinguishing “State A” from “State B” (50% of the data was used for training and the remainder for testing). For each level of coactivity (1, 2, 3, 4, or 5 assemblies), performance was averaged over 5 different “State A” rasters and 4 unique “State B” rasters for each “State A” raster. As a control, the dark gray trace shows mean classifier performance when “State B” was replaced with a second randomly generated template containing 0 inserted assemblies. Inset shows the activity level of each neuron in “State A” plotted against its activity level in “State B.” **(C)** The classifier, trained on the original “State A” and “State B” rasters, performed at chance levels when tested on swap-shuffled versions of the “State A” and “State B” rasters. Swap shuffling each raster destroys correlated patterns of activity. **(D)** Scatter plot comparing accuracy of a linear SVM classifier to the optimal performance of our neural network classifier. In both cases, 50% of the data was used for training and the remainder for testing. Each dot represents the average of 4 simulations using the indicated number of inserted assemblies. **(E)** Scatter plot comparing accuracy of classification using logistic regression to the optimal performance of our neural network classifier. In both cases, 50% of the data was used for training and the remainder for testing. Each dot represents the average of 4 simulations using the indicated number of inserted assemblies. **(F)** We generated synthetic datasets which differ in activity levels. The “State A” rasters were as described in panel A. In this case, “State B” rasters were generated by transferring a proportion of the activity from the first 50 neurons in each State A raster to neurons 51–100. In this manner, half the neurons increased their activity in “State B” compared to “State A”; the other half decreased. The proportion of activity transferred was determined by randomly drawing a number with an upper bound ranging from 5% to 50%. **(G)** We used 50% of the data to train a neural network classifier to distinguish “State A” from “State B.” The remaining data were used for testing. Insert shows the activity level of each neuron in “State A” plotted against its activity in “State B.” The color of the trace indicates the max % of activity that was shifted from neurons 1–50 to neurons 51–100 when generating the State B raster. **(H)** Swap shuffling each raster (preserving differences in the activity levels of individual neurons between States A and B while disrupting correlations) does not decrease classification accuracy. **(I)** Scatter plot comparing the accuracy of a linear SVM classifier to the optimal performance of our neural network classifier. For both classifiers, 50% of the data was used for training and the remainder for testing. Each dot represents the average of 4 simulations, and the color indicates the maximum amount of activity transferred from neurons 1–50 to neurons 51–100. **(J)** Scatter plot comparing the accuracy of logistic regression to the optimal performance of our neural network classifier. For both classifiers, 50% of the data was used for training and the remainder for testing. Each dot represents the average of 4 simulations, and the color indicates the maximum amount of activity transferred from neurons 1–50 to neurons 51–100. Data used to generate this figure can be found in the Supporting information Excel spreadsheet (S1 Data). SVM, support vector machine; LM, linear model (logistic regression).

We first confirmed that swap shuffling preserves information encoded by activity levels, while disrupting the ability of correlated activity to transmit information. For this, we created synthetic datasets, swap shuffled them (within each condition), and then measured classifier performance (**Fig 4**). Specifically, we generated different types of synthetic datasets, which encoded 2 different “behavioral states” (State A or State B) via correlations or activity levels. In all cases, we first created a raster for “State A” by randomly assigning the activity of 100 neurons so that the total activity in each frame oscillated around a mean level. To encode “State B” using correlations, we started with the original “State A” raster, but shifted the activity of individual neurons in time, to create between 1 and 5 cell assemblies composed of coactive neurons. Specifically, each assembly contained 8 neurons; whenever one of these neurons was active, we probabilistically shifted the activity of other neurons in that assembly, so that they would be coactive in the same frame (**Fig 4A**). Notably, the activity level of every neuron was the same in the original “State A” raster, and these newly created “State B” rasters. State B was distinguished simply by an increase in correlated activity. (When we shifted the activity of one neuron in an assembly into a frame, we swapped its activity with that of another neuron that was outside of the assembly; as a result, the total activity in each frame was unchanged).

After training, our neural network classifiers were able to distinguish between “State A” and “State B” (**Fig 4B**). This confirms that this neural network can classify 2 different behaviors even when they are encoded entirely by changes in correlated activity, not by any differences in activity levels. Furthermore, when we swap shuffled the “State B” raster to eliminate these correlations, classifier performance was reduced to chance levels (**Fig 4C**). (Note that in **Fig 2**, we swap shuffled across the entire experiment, eliminating differences in activity between conditions; by contrast, here, we swap shuffled just the State B raster, thereby preserving differences in activity between State A and State B, but removing correlated patterns of activity). Notably, when we used optimal linear classifiers to distinguish between “State A” activity and “State B” activity, either a support vector machine (**Fig 4D**) or logistic regression (**Fig 4E**) we observed chance performance, demonstrating that our neural network classifier was superior for distinguishing states which differ in only the temporal patterning of activity (and not in the activity levels of individual neurons).

Next, we tested the ability of our neural networks to classify “State A” versus “State B” utilizing differences in activity levels. For this, we again started with a random “State A” raster. Then, to generate a “State B” raster, we shifted a proportion of activity from neurons 1 to 50 to neurons 51 to 100 (**Fig 4F**). Thus, in these synthetic datasets, neurons 1 to 50 have higher activity in “State A,” whereas neurons 51–100 have higher activity during “State B.” We varied the proportion of activity that was transferred between neurons. Once again, after training, our neural networks could classify activity patterns corresponding to “State A” versus “State B” (**Fig 4G**). However, in this case, swap shuffling “State B” rasters did not disrupt classifier performance (**Fig 4H**). This confirms that our neural networks classifiers can detect encoding that is based on either activity levels or correlated activity. Swap shuffling a dataset (within one behavioral state) will remove information encoded by correlated activity without disrupting encoding via the activity levels of individual neurons. Notably, both linear support vector machines (SVMs) and logistic regression performed similarly to our neural network for all levels of activity modulation (**Fig 4I and 4J**). Thus, our neural network is specifically superior to optimal linear classifiers at distinguishing states which differ in only the temporal patterning of network activity.

To understand how the neural network distinguishes activity states, we examined the output weights of each hidden unit that resulted from training the classifier. We first examined classifiers trained on the datasets generated by modulating the activity levels of neurons. These datasets were designed so that activity levels for neurons 1:50 would be higher in State A than State B, whereas activity levels for neurons 51:100 were higher in State B (**S3A Fig**). The neural networks were trained to classify States A and B as “1” and “0,” respectively. We noted that neurons 51:100 (increased in “State B”) tended to be connected to hidden units that learned strongly negative output weights during training, whereas neurons 1:50 were more likely to be connected to units with strong positive weights (**S3B and S3C Fig**). After sorting based on output weights, we binned the hidden units into groups of 50 and calculated the likelihood that each neuron was connected to hidden units within a given bin. We defined 2 vectors corresponding to the probabilities of being connected to the hidden units with the most negative or most positive output weights (referred to as the negative or positive ensemble, respectively; **S3D Fig**). Then, we calculated the activity of each subnetwork in frames corresponding to “State A” or “State B.” As expected, the activity of the negative ensemble was higher in State B, whereas activity of the positive ensemble was higher in State A (**S3E and S3F Fig**; blue line, right panels). Thus, over training, the classifier learns to assign positive weights to hidden units that receive the bulk of their input from neurons whose activity increases during frames which should be classified as “1.” Conversely, training assigns negative output weights to hidden units that receive the bulk of their input from neurons that decrease their activity during these frames (and increase their activity during frames which should be classified as “0”).

The preceding intuition does not explain how neural network classifiers might distinguish states which differ in the temporal patterning of activity, but not in neuronal activity levels. To address this question, we focused on our synthetic datasets that were generated by inserting patterns of correlated activity while holding activity levels constant (**S4A Fig**). Again, we examined the output weights assigned to hidden units during training. Here, we observed 2 clear, but seemingly contradictory, patterns. In some cases, the coactive neurons which participated in the inserted assemblies were more likely to be connected to hidden units with strong negative output weights. Conversely, in other cases, these coactive neurons were more likely to be connected to hidden units with strong positive output weights (**S4B and S4D Fig**). As before, we calculated vectors corresponding to the probability that each neuron was connected to hidden units assigned strong negative or positive weights during training to define negative and positive subnetworks. In Example 1, in which the coactive neurons are more likely to be connected to negatively weighted hidden units, activity of the negative subnetwork increases in State B (which contains inserted patterns of activity) (**S4C Fig**). This is in direct contrast to Example 2, in which the coactive neurons are more likely to be connected to positively weighted hidden units. In Example 2, we observed increased activity of the positive subnetwork during “State B” (**S4E Fig**).

How might neural network classifiers utilizing apparently contradictory strategies perform at above chance levels when classifying datasets which differ only in patterns of correlations (not in activity levels)? To understand how this works, consider the example in which there are 2 states (corresponding to output labels 0 and 1) and 2 neurons. In state 0, the 2 neurons are always coactive. In state 1, the 2 neurons are always active at different times. The level of activity (*f*) is the same in both conditions. Thus, for logistic regression, the output weight of each of these 2 neurons would be 0, and an optimal linear classifier based on logistic regression would not be able to perform this classification using the activity of these 2 neurons. However, a neural network classifier with a hidden layer could learn a variety of strategies to classify these 2 states. For example, suppose these 2 neurons converge onto a single hidden layer unit with a positive output weight. Then, time points in which one or both of these neurons are active will be classified as state 1. Because that hidden unit will be active on 2*f* frames in state 1 versus only *f* frames in state 0, this would lead to above-chance performance (frames in which neither neuron is active would be randomly classified as state 0 or 1 with equal probability). Next, suppose that there are additional neurons that have similar levels of activity in both states, and consider 2 hidden units. The first hidden unit receives input from the 2 neurons which are always coactive in state 0 and active out of phase in state 1. The second hidden unit averages activity of the other neurons. In this scenario, if the first and second hidden units have appropriately tuned negative and positive output weights, respectively, then negatively weighted output from the first hidden unit will only exceed positive weighted output from the second hidden unit when the original 2 neurons are coactive, leading to detection of state 0. When only one of the original 2 neurons is active (or neither one is active), the net output would be positive. This strategy will also lead to above-chance performance. As these examples illustrate, a neural network with a hidden layer can learn a variety of strategies that use correlated activity to classify 2 states, even when the activity levels of the individual neurons do not differ between the 2 states. In particular, in some strategies, the correlated neurons connect to hidden units which promote detection of the state in which they are coactive, whereas in others, they provide input to hidden units with the opposite output weight. As is hopefully clear, there is no single explanation for how neural networks successfully classify these types of datasets, but they can exploit multiple strategies to distinguish datasets that differ in patterns of correlated activity, and thereby outperform optimal linear classifiers.

In particular, once training is complete, the hidden layer in the neural network classifier is superfluous, and the classifier could be reexpressed as a logistical classifier. However, the hidden layer plays a key role in training. Specifically, the learning rule operates on the weights from each hidden unit (not weights from the input neurons). Each hidden unit corresponds to an ensemble of input neurons. Thus, the learning rule effectively identifies groups of neurons whose summed activity correlates with the condition, rather than individual neurons which correlate with the condition. This is what enables the neural network to distinguish conditions which differ in coactivity but not the activity of individual neurons.

### SHARC shuffling preserves patterns of correlated activity

**Fig 5:**
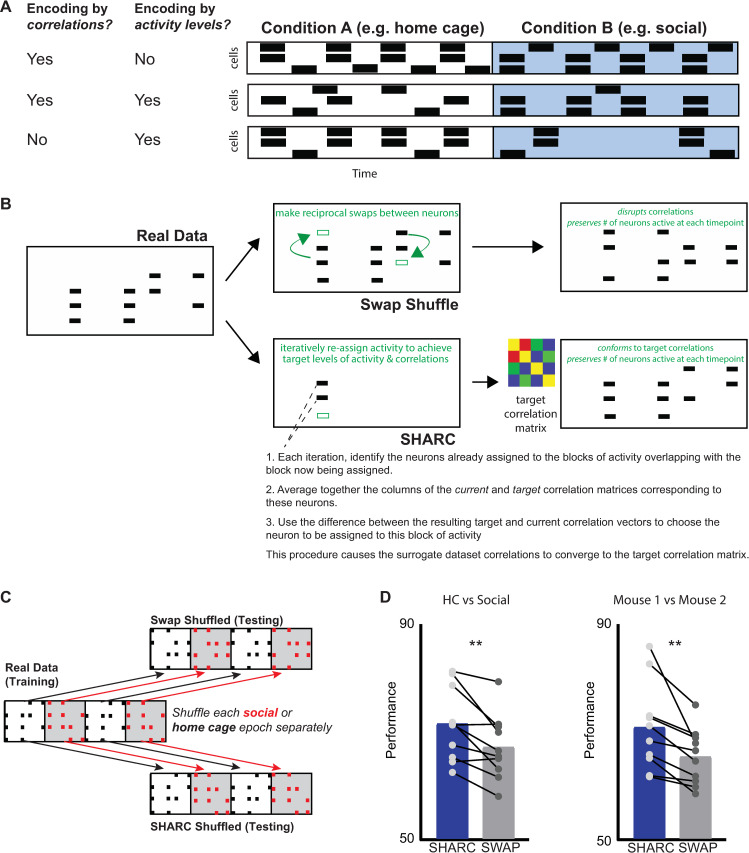
Correlations transmit additional information beyond that conveyed by changes in activity levels. **(A)** Cartoon illustrating that information about behavior may be encoded through changes in activity levels, correlations between neurons, or both. **(B)** To disentangle the roles of activity levels and correlations in transmitting information, we used 2 different methods to create shuffled (surrogate) datasets which preserve changes in activity levels, but either do or do not preserve patterns of correlations. We made random, reciprocal swaps of activity between neurons to generate surrogate datasets which maintained network activity in each frame as well as the number of blocks of activity for each neuron. However, this swap shuffling destroyed the correlation structure. In a second set of surrogate datasets, we used SHARC to iteratively generate surrogates in which the correlation structure was also maintained. **(C)** To maintain behavior-specific changes in activity levels and correlations that are associated with the 2 behavioral conditions, we swap shuffled or performed SHARC separately for each behavioral epoch, then concatenated the 9 resulting surrogate subrasters to create each surrogate dataset. **(D)** We trained a neural network classifier (using input connection probability = 0.3) on each real dataset, then tested that classifier on swap or SHARC-shuffled surrogate datasets generated from that real dataset. Accuracy was significantly higher for the SHARC-shuffled surrogates, which maintain the behaviorally modulated correlations found in the original dataset. Left: accuracy for surrogate datasets in classifying periods of HC vs. SOC behavior = 71 +/− 2 (SHARC) vs. 67 +/− 2% (swap), *p* = 0.005, paired *t* test. Right: accuracy for surrogate datasets in classifying interaction with Mouse 1 vs. interaction with Mouse 2: 71 +/− 3% (SHARC) vs. 65 +/− 2% (swap), *p* = 0.007, paired *t* test. *n* = 10 mice. Data used to generate this figure can be found in the Supporting information Excel spreadsheet (S1 Data). HC, home cage; SHARC, SHuffling Activity to Re-arrange Correlations; SOC, social.

Before applying this same approach to our actual datasets, we also wanted to create a method for shuffling that preserves patterns of correlated activity (**Fig 5A**). For this, we used a method that we published previously: SHuffling Activity to Re-arrange Correlations or SHARC [24]. Like swap shuffling, SHARC also reassigns blocks of activity between neurons, but rather than doing so randomly, it instead follows an algorithm to achieve a target correlation matrix (in this case, the original correlation matrix) (**Fig 5B and 5C**). The full details of SHARC are presented in the Materials and methods section. Briefly, on each iteration, we randomly select one block of activity to be assigned to a new neuron. Instead of choosing the new neuron randomly, we first compute the difference between the target correlation matrix and the correlation matrix of the partially reconstructed surrogate dataset. Then, we assign the block of activity to the neuron which will optimally reduce this difference. Finally, to maintain the mean activity level of each neuron, there is also an absolute limit on how many blocks of activity can be reassigned to each neuron. Notably, when we applied SHARC shuffling to our previously described synthetic datasets, it visibly preserved assemblies of coactive neurons and did not disrupt the ability of neural network classifiers to distinguish between “State A” and “State B” based on either activity levels or correlated activity (**S2 Fig**).

### Patterns of correlated activity contribute to classifier performance

Having confirmed that we can swap or SHARC shuffle individual activity during individual epochs to preserve activity levels while either disrupting or preserving correlated activity, we now used this approach to quantitatively measure the encoding of social behavior by activity levels versus correlated activity in our actual datasets. Specifically, we swap or SHARC shuffled each social or home cage subraster separately (again this is in contrast to **Fig 2**, in which we shuffled data across the entire experiment). Then, we concatenated these shuffled subrasters to create swap or SHARC-shuffled surrogate datasets that preserve behaviorally specific levels of activity, while disrupting or preserving patterns of correlated activity, respectively.

Both swap and SHARC-shuffled surrogate datasets preserved levels of activity observed during either social interaction or periods when mice were alone in their home cages. Specifically, we computed the correlation between vectors in which each element represents the activity level of one neuron during one behavioral condition and quantified the correlation (similarity) between each real and surrogate dataset. For swap-shuffled surrogate datasets, the similarity of activity levels (compared to real data) was 0.89 +/− 0.02 in the home cage and 0.82 +/− 0.04 during social interaction. For SHARC-shuffled surrogate datasets, the similarity of activity levels (compared to real data) was 0.88 +/− 0.03 in the home cage and 0.86 +/− 0.03 during social interaction (*n* = 10 mice/datasets). We also computed the similarity of the pattern of correlations between each surrogate dataset and the corresponding real dataset. In this case, only SHARC-shuffled surrogate datasets preserved patterns of correlations. For swap-shuffled surrogate datasets, the similarity of correlations to the real data was 0.01 +/− 0.01 in the home cage and 0.03 +/− 0.01 during social interaction. For SHARC-shuffled surrogate datasets, the similarity was 0.50 +/− 0.05 in home cage and 0.55 +/− 0.03 during social interaction.

We then trained classifiers on each dataset and tested each classifier using either swap or SHARC-shuffled surrogate datasets generated from the same dataset using for training (**Fig 5C**). Classifiers performed better than chance when tested with either surrogate dataset indicating that changes in activity levels encode behavioral information, making it possible to distinguish bouts of social interaction from periods when a mouse is alone in its home cage. However, classifier accuracy was significantly higher for SHARC-shuffled surrogates datasets than for swap-shuffled ones (**Fig 5D**, left; accuracy for classifying home cage versus social = 71 +/− 2% for SHARC versus 67 +/− 2% for swap-shuffled datasets, *p* = 0.005, paired *t* test).

### Patterns of correlated activity also help encode mouse identity

We wondered whether we could use this approach to study how correlations contribute to encoding of additional information besides just whether or not a mouse was engaged in social interaction. In our behavioral design, the subject mouse interacted with one mouse for 2 epochs and with a second mouse during 2 different epochs. Therefore, we also trained neural networks to classify whether the subject mouse was interacting with Mouse 1 versus Mouse 2. Again, a neural network classifier was able to perform this classification at above-chance levels, and performance was higher when the classifier was tested using SHARC-shuffled surrogate datasets than when tested using swap-shuffled surrogate datasets (**Fig 5D**, right: accuracy for classifying Mouse 1 versus Mouse 2 = 71 +/− 3% for SHARC versus 65 +/− 2% for swap-shuffled datasets, *p* = 0.007, paired *t* test). This shows that behaviorally modulated patterns of correlated activity transmit additional information, beyond what is readily decodable from activity levels alone. This is important because whereas differences in motor activity or arousal may contribute to distinct activity patterns observed during home cage exploration and social interaction, differences in activity observed during interactions with different mice are not related to nonspecific factors, e.g., the general presence of odors or sounds, but rather must be mouse specific, and thus carry social information.

### Combinations of coactive neurons occur in a behaviorally specific manner

Interestingly, neural networks perform classification better for connection probabilities approximately 0.2 to 0.4 than for connection probabilities <0.1. When the connection probability is low, each hidden unit receives input from individual prefrontal neurons or small groups of neurons. By contrast, when the input probability is higher, hidden units receive input from larger groups of prefrontal neurons. This suggests that training proceeds more efficiently when the network represents information about social versus home cage behavior using multineuron combinations, instead of activity within individual neurons. Therefore, as a proof of concept, we directly examined whether 3-neuron patterns of coactivity occur in a behaviorally specific manner. We examined 3-neuron combinations because they measure network structure beyond pairwise correlations and are the building blocks of larger combinations. One could in principle analyze larger combinations, but because of the limited numbers of neurons and frames in our datasets, there is not always adequate statistical power to resolve larger combinations, i.e., to identify large combinations which occur more often than expected by chance.

First, we quantified how often each possible 3-neuron combination occurred in real datasets. Then, we calculated how often each of these combinations occurred in datasets that had been swap shuffled (across the entirety of the dataset). For each real dataset, we constructed 1,000 swap-shuffled datasets and identified “enriched combinations,” which occurred more often in real datasets than expected by chance, i.e., than in 95% of swap-shuffled surrogate datasets. Finally, we quantified how many enriched combinations occurred exclusively during social or home cage epochs. Combinations could appear to be behaviorally specific simply because they only occurred at a single time point. Therefore, we also restricted our analysis to enriched combinations which occurred during multiple distinct bouts of social interaction and/or matched sets of intervals during home cage epochs. Many of these repetitively occurring enriched combinations were behaviorally specific: 43.5% occurred during social interaction, 26.5% during home cage epochs, and 30% during both conditions.

The behaviorally selective occurrence of enriched combinations could in principle reflect changes in single neuron activity (i.e., neurons that form a social combination are only active during the social condition) and/or changes in correlations (i.e., neurons are active in both conditions but only coactive during social behavior). We defined specific enrichment as combinations which occurred more often in real data than in 95% of swap-shuffled surrogate datasets for one behavioral context and less in real data than in 50% of swap-shuffled surrogate datasets for the other context. We then identified instances in which a social and nonsocial 3-neuron combination overlapped in 2 out of 3 neurons. In the majority of these cases, the neuron which was part of a 3-neuron combination (triplet) in one behavior but left out of the overlapping triplet in the opposing behavior was instead part of a different 3-neuron combination that was enriched during the second behavior (**S5 Fig**). Thus, the specificity of a combination of coactive neurons for social versus nonsocial behavior does not occur simply because some neurons were only active during one condition, but rather reflects the dynamic reorganization of patterns formed by neurons which are active in both conditions, i.e., changes in correlations. This behaviorally specific occurrence of multineuron patterns of coactivity represents the substrate through which correlations can add to the behavioral information transmitted by neuronal ensembles.

### Socially enriched combinations are deficient in *Shank3* KO mice

**Fig 6:**
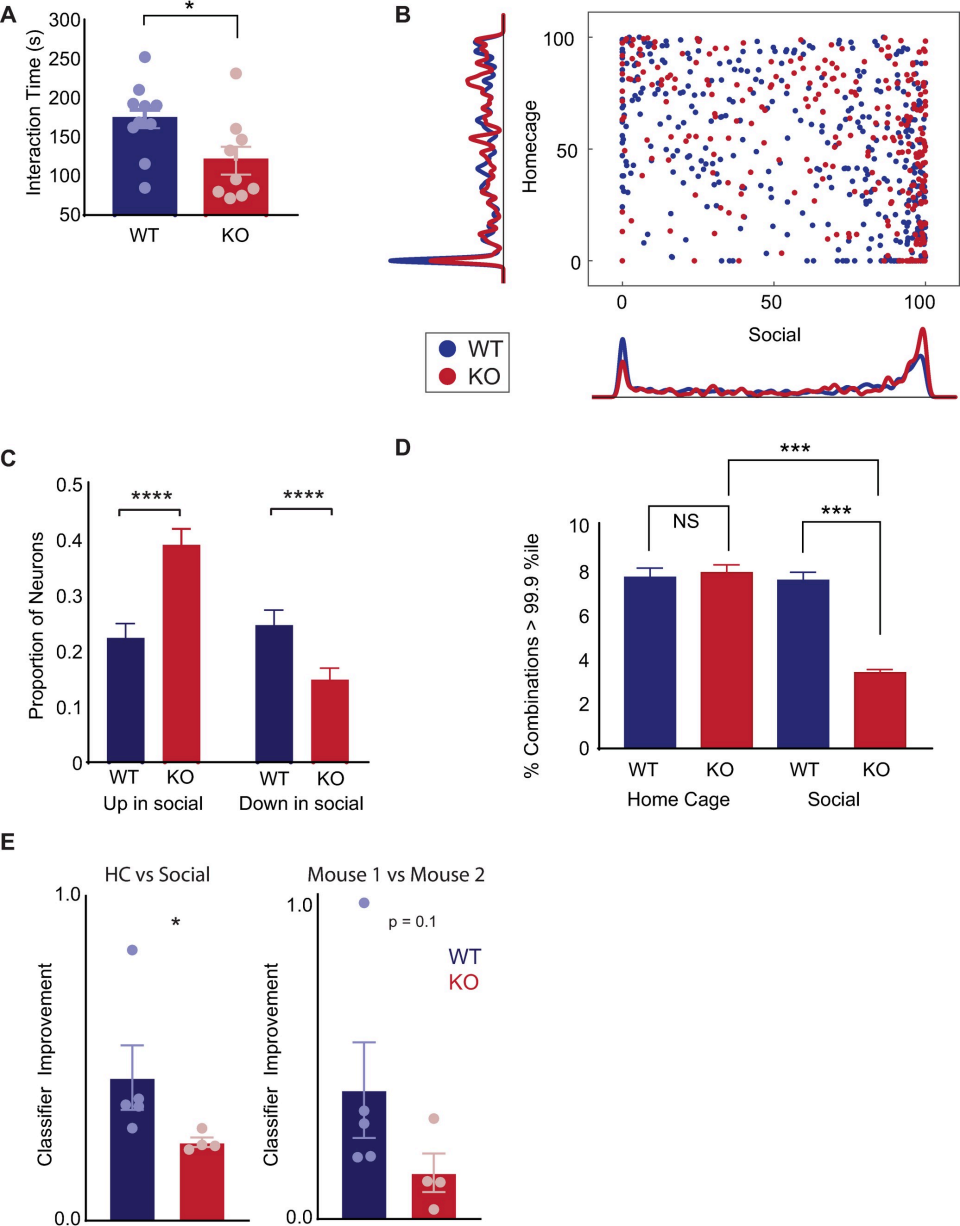
*Shank3* KO mice have disorganized ensembles for which correlations fail to enhance the transmission of information about social behavior. **(A)** The mean time that *Shank3* KO mice or WT littermates spend interacting with a novel juvenile mouse of the same sex during a 5-minute assay. Data have been pooled from 8 unimplanted WT mice as well as the 5 implanted WT mice used for microendoscopic imaging and 5 unimplanted KO mice in addition to the 4 implanted mice used for imaging. For implanted mice, we used the average of interaction time for the 2 novel mice. Pooled data showed decreased interaction in KO mice (173 +/− 12 seconds vs. 120 +/− 18 seconds for WT and KO respectively, *p* < 0.05, *t* test). The unimplanted cohort alone shows a similar significant decrease in interaction time for KO mice (165 +/− 15 seconds vs. 110 +/− 16 seconds for WT and KO respectively, *p* < 0.05, *t* test). In the implanted cohort, there was a similar trend toward decreased interaction for KO mice (186 +/− 20 seconds vs. 133 +/− 37 seconds for WT and KO respectively, *p* = 0.21, *t* test). **(B)** (Similar to **Fig 1D**). Scatter plot showing the activity of each neuron during each behavioral condition, expressed as a percentile relative to a null distribution generated by circularly shuffling that neuron’s activity. Activity levels during social interaction or while the mouse was alone in its HC are plotted on the x-axis and y-axis, respectively. Kernel density plots along the axes indicate the fraction of neurons whose activity was at a given percentile of the null distribution. Neurons with activity >90th percentile of shuffled datasets (green dotted line) were considered to be positively modulated, whereas neurons with activity <10th percentile (green dotted line) were considered to be negatively modulated during each behavior. Data are plotted for *Shank3* KO mice (red) and WT littermates (blue) (mean percentile of activity during social interaction, WT: 50 +/− 2 percentile; KO: 64 +/− 2 percentile; *p* < 0.0001 by 2-sample *t* test; mean percentile of activity during HC, WT: 47 +/− 2 percentile; KO: 51 +/− 2 percentile; *p* = 0.1, *t* test). **(C)** Bar graph showing the fraction of neurons whose activity was positively or negatively modulated (>90th percentile or <10th percentile) during social interaction. The proportion of neurons which increased activity during social interaction was significantly greater in KO mice (22% in WT vs. 39% in KO, chi-squared = 17.7, *p* < 0.0001), whereas the down-regulated ensemble was significantly smaller in KO mice (25% in WT vs. 15% in KO, chi-squared test, 8.2, *p* < 0.005). Error bars denote the binomial SEM algebraically derived from total number of neurons and the proportion that were modulated in the specified direction. **(D)** The proportion of 3-neuron combinations occurring during HC exploration that are enriched >99.9th percentile compared to swap-shuffled datasets was similar across WT (blue; 7.6%) and KO (red; 7.8%) mice. By contrast, the proportion of 3-neuron combinations occurring during social interaction that are enriched >99.9th percentile compared to swap-shuffled datasets was 7.5% in WT compared to only 3.4% in KO mice (total number of HC combinations: 4,187 in 5 WT mice and 5,878 in 4 KO mice; total number of social combinations: 5,487 in 5 WT mice and 16,326 in 4 KO mice). We have plotted this data as a bar graph showing the fraction of these combinations that were specifically enriched above the 99.9th percentile (chi-squared = 165, *p* < 0.0001). Error bars denote the SEM algebraically derived from the binomial distribution, the number of 3-neuron combinations in each condition, and the proportion of those combinations that were enriched. **(E)** Bar graph showing how much better classifiers trained on real datasets perform when tested on SHARC-shuffled datasets, compared to their performance when tested on swap-shuffled datasets, i.e., **(PerformanceSHARC – PerformanceSwap)/(PerformanceSwap − 0.5)**. Each datapoint represents a classifier from one mouse. Left: The increase in performance was significantly larger in WT mice (blue) than KO mice (right) for classification of HC periods (HC) vs. epochs of social interaction (Social), WT: 0.44 +/− 0.10 vs. KO: 0.24 +/− 0.01, *p* = 0.016, Mann–Whitney. Right: The increase in performance was nonsignificantly larger in WT mice (blue) than KO mice (right) for classification of interactions with Mouse 1 vs. those with Mouse 2, WT: 0.41 +/− 0.15 vs. KO: 0.14 +/− 0.06, *p* = 0.11, Mann–Whitney. Combined analysis of both classifications (HC vs. Soc and Mouse 1 vs. Mouse 2) shows a significant effect of genotype: *p* < 0.05 by 2-way ANOVA. Data used to generate this figure can be found in the Supporting information Excel spreadsheet (S1 Data). HC, home cage; KO, knockout; SHARC, SHuffling Activity to Re-arrange Correlations; WT, wild-type.

We were curious whether there might be conditions under which these phenomena—the occurrence of multineuron combinations of coactivity during social behavior and the ability of correlated activity to enhance the transmission of information about social behavior—might be impaired. To explore this, we performed microendoscopic GCaMP imaging in mice lacking the autism-associated gene *Shank3* [25–27]. These mice have been extensively studied as models of Phelan–McDermid syndrome, which often includes autism as a clinical feature. *Shank3*^−/−^ (KO) mice are known to have social deficits, and indeed, we found that compared to WT littermates, they spend significantly less time interacting with novel juvenile mice (**Fig 6A**).

We compared patterns of prefrontal activity in *Shank3* KO mice and their WT littermates. As in WT mice, many prefrontal neurons in *Shank3* KO mice either increase or decrease activity during social interaction. However, compared to WT mice, the fraction of neurons whose activity increases during social interaction was significantly higher, whereas the fraction whose activity decreases was significantly lower (**Fig 6B and 6C**; 22% of 260 WT neurons versus 39% of 290 KO neurons increased activity above the 90th percentile of shuffled data during social interaction, chi squared = 17.7, *p* < 0.0001; 25% of WT versus 15% of KO neurons decreased activity below the 10th percentile of shuffled data during social interaction, chi squared = 8.2, *p* < 0.0001). Thus, *Shank3* KO mice recruit abnormal neuronal ensembles during social behavior. We hypothesized that this might reflect a network-level disorganization that affects the normal patterning of activity during social behavior.

Indeed, we found that in KO mice, a significantly smaller fraction of the 3-neuron combinations observed during social interaction was strongly enriched, i.e., occur more often in actual data than in 99.9% of swap-shuffled surrogate datasets (**Fig 6D**). This suggests that even though more neurons (i.e., larger ensembles) were recruited during social behavior in KO mice, these may have been less well organized, such that the occurrence of socially enriched patterns of activity is obscured by “noise,” i.e., patterns formed by the chance overlap of activity between neurons that fire in a largely independent fashion. Notably, this deficiency was specific for social interaction. The fraction of 3-neuron combinations that were strongly enriched during home cage exploration (in comparison to swap-shuffled surrogate datasets) was similar in WT and KO mice (**Fig 6D**).

### The ability of correlated activity to contribute to the encoding of social behavior is disrupted in *Shank3* KO mice

The preceding shows that even though social behavior robustly recruits neuronal ensembles in *Shank3* KO mice, the organization of these ensembles into multineuron combinations is disorganized. This suggests that the ability of patterns of correlated activity to encode information about social behavior may be impaired in these mice. To test this, we examined whether correlated activity contributes to the transmission of information about social behavior in *Shank3* KO mice. As before, we generated swap and SHARC-shuffled surrogate datasets, then tested the ability of classifiers trained on the original datasets (from *Shank3* KO mice) to classify activity associated with behavior during social interaction versus in home cage. We then compared the degree to which classifier accuracy was improved when testing SHARC-shuffled surrogate datasets compared to testing swap-shuffled ones. In datasets from *Shank3* KO mice, we observed a significantly smaller improvement in classifier accuracy for SHARC versus swap-shuffled datasets (**Fig 6E**), indicating a diminished role for correlated activity in encoding (relative improvement for classifying home cage versus social: 0.44 +/− 0.10 in WT versus 0.24 +/− 0.01 in KO; relative improvement for classifying whether the subject was interacting with Mouse 1 versus Mouse 2: 0.41 +/− 0.15 in WT versus 0.14 +/− 0.06 in KO; 2-way ANOVA using classification type and genotype as factors: significant for genotype, *p* < 0.05, no effect of classification type, *p* = 0.58, or interaction, *p* = 0.76; the overall classifier performance, averaged over both home cage versus social and Mouse 1 versus Mouse 2 discriminations, in WT was 66.6 +/− 2.7% for swap-shuffled versus 75.5 +/− 2.9% for SHARC-shuffled; in KO, the overall classifier performance was 67.1+/− 2.2% for swap-shuffled versus 70.2 +/− 1.7% for SHARC-shuffled datasets) Thus, in *Shank3* KO mice, the multineuron patterns of coactivity which normally occur during social behavior are obscured by noise, and as a result, patterns of correlated activity contribute less information about social behavior.

## Discussion

During complex behaviors, the brain can use many strategies to represent information about the external environment and internal state of the organism. The term “ensemble” is often used to refer to a group of neurons whose activity is similarly modulated (either increased or decreased) during specific behaviors [1,28–32]. It is generally accepted that ensembles transmit behavioral information via changes in the activity levels of their constituent neurons. On the other hand, many studies have also shown that correlations between neurons can change during specific behaviors [3,11] or behavioral states [33–35]. Importantly, correlations reflect changes in coactivity which exceed those expected to occur simply because of changes in the activity levels of the individual neurons [6], i.e., when an ensemble becomes more active, correlations between the neurons in that ensemble could go up, down, or remain unchanged. By optimizing synaptic interactions such as temporal summation, changes in correlated activity could potentially act to enhance the behavioral information transmitted by changes in ensemble activity or transmit additional information.

The role of correlations in information transmission has been studied extensively in the isolated retina [36]. In the cortex, noise correlations—correlations in trial-to-trial variability across neurons that are thought to be unrelated to the variable of interest—have also been extensively studied [13,15,16]. Noise correlations can enhance information transmission, presumably by helping to disambiguate changes in activity levels due to the variable of interest (signal) from those attributable to other causes (noise). However, the ability of correlations to transmit information themselves, by changing as a function of the variable being encoded, has been less well studied. In particular, it is not clear whether changes in correlations encode additional information beyond what is transmitted by changes in activity levels, and if so, whether this reflects the behaviorally specific occurrence of patterns of correlated activity/coactivity.

Here, we addressed this question using microendoscopic GCaMP imaging to measure activity from many (approximately 40 to 100) prefrontal neurons during social behavior in mice. We used a simple neural network, in which prefrontal neurons provide input, there is one hidden layer, and a single output unit classifies social versus nonsocial behavior, to quantify how well prefrontal ensembles would transmit information about social behavior to downstream neurons. We used multiple approaches to disentangle the respective contributions of changes in activity levels versus correlated activity to the encoding of social behavior. First, whereas previous studies have compared information transmission by real datasets versus by shuffled ones [16], we extended a method we previously published [24] to nonrandomly shuffle datasets in order to preserve both behaviorally modulated correlations and ensemble activity. This enabled us to compare the amount of information about social behavior transmitted by either SHARC-shuffled surrogate datasets or randomly shuffled surrogates which preserved ensemble activity but not correlations. In this way, we found that correlated activity enhances the amount of information that prefrontal ensembles transmit about social behavior. Notably, we used neural networks to classify periods when a subject mouse was alone in its home cage versus interacting with another mouse and separate classifiers to determine whether the subject was interacting with test Mouse 1 versus test Mouse 2. We found that correlated activity normally enhances the encoding of both types of information.

Second, when we examined connections within neural network classifiers, we found that prefrontal neurons which serve to detect social behavior increase their correlations during social behavior (whereas “neutral” neurons and neurons which detect nonsocial behavior do not). The fact that during social behavior the home cage ensemble increases activity (but not correlated activity), whereas the social ensemble increases activity and correlated activity can be conceptualized as implementing a computation. Specifically, the neural network classifier is effectively measuring the difference between the amount of coactivity that reflects correlated activity specifically associated with social behavior (coactivity of the social ensemble) and the amount that occurs just by chance based on the overall level of activity in the network (coactivity of the home cage ensemble). Third, we found that combinations of coactive neurons, which occur more often than expected based on the activity levels of the constituent neurons, manifest in a behaviorally specific manner. Positive correlations measure neuronal coactivity that occurs more often than expected based on the chance overlap of activity between neurons. Thus, in accordance with our finding that social behavior modulates correlations, we found that multineuron patterns of coactivity which occur more often than expected by chance are behaviorally specific. We also directly examined these behaviorally specific and statistically enriched combinations of coactive neurons. We found that they tend to be composed of neurons which are active in both conditions but only coactive in one, rather that neurons which are only active in one condition. Thus, we used multiple new approaches (comparing classifier accuracy for SHARC versus swap-shuffled datasets, measuring changes in correlations for neurons in ensembles derived from neural network classifiers, and linking statistically enriched patterns of coactivity to specific behaviors) to show exactly how dynamic correlations encode information about social behavior. Critically, this does not imply that the brain actually uses this information for decoding. However, the fact that we found that a very simple neural network based on biologically realistic mechanisms can decode social information using correlated activity suggests that the brain could readily use this strategy.

Interestingly, statistically enriched patterns of coactivity were specifically deficient during social behavior in mice lacking the autism-associated gene *Shank3*. Accordingly, in *Shank3* KO mice, we found a reduction in the ability of surrogate datasets which preserve behaviorally modulated correlations to transmit more information about social behavior compared to randomly shuffled datasets which only preserved ensemble activity. This shows that the ability of correlated activity to enhance the transmission of information about social behavior is not automatic and can in fact be disrupted in pathological states.

Social behavior (and home cage exploration) are both presumably composed a number of sub-behaviors, each associated with distinct underlying patterns of neural activity. We did not to examine these sub-behaviors and associated patterns of activity, because this have required using correspondingly fewer frames of neuronal activity for training and testing. It is likely that our classifier utilizes multiple patterns of activity, each of which may be specifically associated with one or more sub-behaviors, to broadly distinguish social interaction from home cage exploration.

Similar to other recent studies [14,15], we have studied activity using binary activity rasters derived from GCaMP imaging. However, an important caveat is that any method of quantifying neural activity has limitations, such that there could be additional ways that neurons encode information which are not well resolved using this approach.

### What is the meaningful size of ensembles in the cortex?

Complex behavior is possible because the brain reliably encodes features pertaining to the external environment as well as the internal state of the organism. These features may be encoded by neuronal ensembles [1,28–32]. What size of ensemble reliably encodes an aspect of behavior? We explored this question by asking what input connection probability would optimize the ability of a downstream network to classify behavior based on input from prefrontal ensembles. Note: Input connections in neural network classifiers do not necessarily correspond to actual connections in the brain—rather they provide information about the size and nature of neuronal ensembles across which information should be combined to most efficiently decode behavior. Peak classifier performance occurred for connection probabilities approximately 0.2 to 0.3. Performance was markedly lower when the connection probability was 0.5. This is surprising because a connection probability of 0.5 would maximize the entropy associated with each input connection. Thus, from the standpoint of encoding social behavior, combining activity from 20% to 30% of the input neurons may achieve some synergy that becomes degraded when ensembles are enlarged beyond this size. In the brain, nonrandom network connectivity [37,38] may similarly produce correlated activity/coactivity within ensembles of this size [39,40].

### Combinatorial codes versus sequential patterns of activity

Like many recent studies, we measured population-level activity in the mouse neocortex using genetically encoded calcium indicators. These indicators transduce neuronal spiking on timescales approximately 100 msec. Thus, correlated activity/“coactivity” imply that neurons jointly increase their activity within windows approximately 100 msec and do not necessarily imply synchronous spiking on faster timescales (milliseconds or even tens of miliseconds). At the same time, correlated activity/coactivity on these timescales should be differentiated from sequential activity of neurons observed during the performance of sequential behaviors (i.e., spatial navigation or overtrained tasks) in which the activity of specific neurons corresponds to moving through a specific location or performing a specific portion of a complex task. As discussed above, in the neocortex correlations and coactivity likely reflect recurrent neural network connectivity [39]. By contrast, sequential patterns of neuronal activation can occur simply as a by-product of the arrangement of spatial locations along a trajectory, the stereotyped order in which cues are encountered during a task, etc.

### Relevance to disease states

Interestingly, in *Shank3* KO mice, which exhibit social deficits, the mPFC successfully recruits specific neuronal ensembles during social interaction. However, these ensembles are enlarged, their organization into statistically enriched patterns of coactivity is disrupted, and correlations between neurons fail to enhance the information that these ensembles transmit. Thus, the computational units by which information is processed in the mPFC appears to be inefficient, i.e., social behavioral recruits an abnormally large number of neurons at the expense of the precise temporal patterning of this activity. This central finding is similar to other findings in rodent models of autism at both the single neuron and network levels [21,25,41]. In particular, we found an increase in the recruitment of prefrontal neurons during social interaction. This mirrors a recent study which found hyperdynamic response to whisker stimulation in the same mice [25], possibly reflecting GABAergic circuit dysfunction and/or homeostatic compensations [42]. (Note: These findings cannot be ascribed simply to the fact that *Shank3* KO mice spend less time engaged in social interaction than their WT littermates; reduced interaction time would tend to reduce statistical power and thereby reduce, not increase, the number of neurons that change their activity more than expected by chance.) An important question for future studies is whether these differences we found in *Shank3* KO mice are sex differential.

Increased excitatory activity causing decreased signal-to-noise ratio (SNR) has long been posited to contribute to the pathophysiology of autism [43]. However, the exact nature of “signal” and “noise” and the specific mechanism through which excessive activity degrades the SNR have been unclear. Here, we show how enlarged neural ensemble recruitment by specific behavioral conditions disrupts information transmission by degrading the ratio between statistically meaningful patterns of coactivity (the signal) and the random overlap of activity between neurons (noise). Thus, in KO mice, information is transmitted through a fundamentally less efficient mode of communication which requires the activation of a greater share of the network.

## Materials and methods

All experiments were conducted according to the National Institutes of Health (NIH) guidelines for animal research, and protocols were reviewed and approved by the Institutional Animal Care and Use Committee (IACUC) at the University of California, San Francisco (UCSF; protocol number AN185374).

### Behavior

C57/B6J mice were obtained from Jackson Laboratories (Bar Harbor, ME). We utilized adult mice of either sex housed and bred in the UCSF animal facility. Adult mice were habituated to the room and observer for 3 days prior to test day. All videos were subsequently scored by a blinded observer. For imaging experiments, 5 WT and 4 KO littermates were generated through crosses between *Shank3* heterozygous parents and injected with AAV5.Syn.GCaMP6f.WPRE.SV40. We included an additional 5 WT mice which were injected with AAV5.Syn.GCaMP6m.WPRE.SV40 [44]. Viruses were obtained from Penn Viral Core. Injections and 500 um GRIN lens (Inscopix) implantations were carried out in 8- to 12-week-old mice to express GCamp6f in prefrontal cortical neurons under control of the human Synapsin promotor. Mice were anesthetized with 2% isoflurane and mounted in a stereotactic frame. Craniotomies were made according to stereotaxic coordinates relative to Bregma. Coordinates for injection into mPFC were (in mm relative to Bregma) +1.7 anterior–posterior (AP), –0.3 mediolateral (ML), and –2.75 dorsoventral (DV), and GRIN lenses were implanted at the same AP and ML coordinates to a depth of 2.25. We subsequently attached baseplates for attaching the microendoscope, approximately 4 weeks later depending on GCamp expression. Mice were habituated for 3 days with the scope attached, prior to test day. On test day, mice were habituated with the scope turned on, then imaged in alternating home cage and social epochs. During social epochs, one of 2 novel sex-matched juvenile mouse was introduced to the test mouse’s home cage in sequential order so that there were 2 “novel” epochs, followed by 2 “familiar” epochs interleaved with “home cage” epochs during which the juvenile mice were removed, and the test mouse was free to explore its home cage. The first and last home cage epoch were 10 minutes in length; the others were 5 minutes in length. Each social epoch lasted 10 minutes, but only the first 5 minutes were recorded and scored. During each behavioral epoch, observer was not in the room. Interaction epochs were defined from the moment test mouse first sniffed the juvenile conspecific or object until the test mouse turned away. Videos were recorded using Anymaze and scored by a blinded observer. For the bulk of analysis, we pooled data across 10 WT mice. *Shank3* KO mice were compared only to recordings from WT littermates.

### Image acquisition and segmentation

Images were acquired using an Inscopix nVoke micreoendoscope attached to a laptop computer and synced to a separate video acquisition computer running Anymaze. Frame rate was 20 Hz, and the light power was 0.2 mW. Acquisition was performed using 2 × 2 pixel binning, then subsequently downsampled again by 2.

We segmented neuronal signals using a modified PCA/ICA approach [22,23], modified so that each segment was expressed as a binary ROI consisting of pixels representing a single neuron, i.e., we used the output from the PCA/ICA to identify a set of contiguous pixels which represent a neuron, then averaged fluorescence signals across those pixels. To deconvolve neuronal signals from background neuropil signals, we also subtracted the mean signal from each identified segment from the mean value in pixels surrounding the edge of the segment (we excluded pixels that belonged to another ROI). Signals were subsequently low-pass filtered to remove high-frequency noise using the following MATLAB command: designfilt(“lowpassfir,” “PassbandFrequency,” 0.5, “StopbandFrequency,” .65, “PassbandRipple,” 1, “StopbandAttenuation,” 25). All signal traces shown represent normalized versions of the *dF*/*F*_*0*_ trace, where *F*_*0*_ is estimated by the median value in the surround region. Threshold based event detection was performed on the traces by detecting increases in *dF*/*F*_*0*_ exceeding 3σ over 1 second, then only keeping those events which exceeded a 15σ increase over 2 seconds, and a total area under the curve of 250σ. As there were occasional downward deflections due to surround subtraction, we instituted a final parameter requiring that the peak cross an absolute value of *dF*/*F*_*0*_ = 0.0125. σ is the standard deviation of *dF*/*F*_*0*_, calculated over the least active 50% of the movie. In some cases, these parameters were adjusted slightly to optimize event detection to >95% sensitivity and specificity, based on visual inspection, for each movie. After identifying these events in the GCaMP signal from a cell, the cell was considered “active” during the entire period from the beginning of an event until the GCaMP signal decreased 30% from the peak of the event, up to a maximum of 2 seconds. The peak of the event was defined as the local maximum of the entire event, from the beginning of the event until *dF*/*F*_*0*_ returned to the pre-event baseline value. Calcium traces from segmented neurons were visually inspected, and a small number of segments were removed if they did not appear to represent a single, unduplicated neuron. We restricted further analysis to those mice with 25 or more active neurons. We then created 2D event rasters consisting of detected events for each neuron over the course of the experiment.

### Detection of behaviorally modulated neurons

To determine the response of individual neurons to behavioral context, we averaged the activity of each neuron during frames corresponding to periods of social interaction or to a temporally matched set of frames during the preceding home cage epoch for all analysis. This allowed us to use an identical number of frames for each condition and control for temporal correlations within each behavioral condition. We then created a “null distribution” for each neuron that represents the percent of time active expected in each condition based on chance by circularly shuffling the data 10,000 times. We then compared the activity of each neuron during either social interaction or home cage exploration to this null distribution. Neurons were considered positively modulated if they exceeded the 90th percentile of that observed in circularly shuffled datasets and negatively modulated if the percent of frames that a neuron was active during a given context was below the 10th percentile of observations from circularly shuffled data.

### SHARC

SHARC is an iterative method for generating surrogate datasets. SHARC nonrandomly shuffles blocks of activity within a raster to generate a new (surrogate) raster in which the pairwise correlations between neurons match a target correlation matrix [23]. Here, we apply this previously published method, with modifications to also preserve the activity level in each neuron (**Fig 4B**).

To begin, note that each raster is equivalent to a collection of blocks of activity. Each block of activity is defined by the time at which it begins, its duration, and the neuron which is active. On each iteration, one block of activity is randomly chosen and assigned to a new neuron as follows. Suppose block *i* has been chosen to be reassigned. First, we find all the blocks of activity that overlap with block *i*. Next, we selected the subset of these blocks for which new cell identities had already been assigned. Call this set *X*. Let *r*_*j*_ represent the number of time points over which block *j* ∈ *X* overlaps with block *i*, and let *n*_*j*_ represent the identity of the cell assigned to block *j* ∈ *X*. *L*_*i*_ and *L*_*j*_ are the lengths of blocks *i* and *j*, respectively. Then, we constructed a vector,

$${\vec{P}}_{i}=\sum_{j\in X}\frac{r_{j}}{\sqrt{L_{i}L_{j}}}({\vec{C}}_{n_{j}}-{\vec{C}\prime }_{n_{j}}),$$

where ${\vec{C}}_{n_{j}}$ represents row *j* of the target correlation matrix, i.e., the target correlations between neuron *n*_*j*_ and the other neurons, and ${\vec{C\prime }}_{n_{j}}$ contains the current values of the correlations between neuron *n*_*j*_ and the other neurons based on the blocks of activity that have already been reassigned. This step can be thought of as “guessing” which cell should be assigned to a particular block of activity by first figuring out what other cells are active at the same time, then choosing cells which are strongly correlated with these known active cells. Note that we assign values of ${\vec{P}}_{i}$ (i.e., construct “guesses” about which cell should be active) using the difference between the current correlation matrix (${\vec{C}\prime }_{n_{j}}$) and the target correlation matrix (${\vec{C}}_{n_{j}}$) in order to identify cell pairs for which the current correlation deviates from the target value and force the new correlation matrix to progressively approximately the target correlation matrix.

We set elements of ${\vec{P}}_{i}$ to 0 if the corresponding neuron had already been assigned to a block of activity that overlaps with block *i*, i.e., element *n*_*j*_ of ${\vec{P}}_{i}$ was set to 0 ∀ *j* ∈ *X*. Finally, we assigned block *i* to the neuron corresponding to the maximum value of ${\vec{P}}_{i}$. This can be thought of as choosing the cell that represents the “consensus” based on tallying up all of the “guesses” about which cells “should” be assigned to the block of activity being considered.

When all the elements of ${\vec{P}}_{i}$ were 0, e.g., because there no overlapping blocks of activity have had new cell identities assigned yet, then we chose a cell in order to match the originally observed level of activity. Specifically, after every iteration, we kept a log of the net number of blocks of activity that each neuron had donated or received. We used this vector to create a weighted probability whereby events from neurons which had received a net positive number of blocks were more likely to be chosen to be reassigned. To further ensure that the total number of active events for each neuron in the surrogate dataset was similar to the real dataset, if the difference between the number of blocks gained—lost in the reassignment process exceeded +4 for a particular neuron, then that neurons was no longer eligible to receive additional blocks of activity; similarly blocks of activity belonging to neurons who had lost greater than 3 blocks of activity were no longer eligible be reassigned. As the selection of blocks of activity for reassignment is probabilistic, we first perform a swap shuffle to ensure that all blocks of activity in the original dataset are swapped at least 1 time, then perform a total of 5 reassignments for each block of activity in the source dataset.

We extended this approach to generate surrogate datasets by shuffling data within shorter time windows (i.e., individual behavioral epochs). Here, a discrete set of frames is chosen, corresponding to a subraster of the original raster. By repeating the process described above for each subrasters, then recombining the shuffled subrasters, we generate a complete shuffled dataset.

### Classifier

We designed and trained a neural network to classify behavior (periods when a mouse was alone in its home cage versus engaged in social interaction). This network contained 1,000 units in a hidden layer, each of which received input from specific prefrontal neurons (from the real dataset). Thus, in each frame, the activity of each hidden layer unit was determined by the summed activity of the connected prefrontal neurons. Each hidden layer unit had an output weight that represented the strength of its connection to a single output unit. On each frame, the activity of the output unit was computed as

$$y=\frac{1}{1+e^{-\sum w_{i}x_{i}}},$$

where *w*_*i*_ is the output weight from hidden unit *i* and *x*_*i*_ is the activity of hidden unit *i*.

When we performed training and testing using the same dataset, we divided the dataset into alternating blocks of 500 frames for training versus testing (in other cases, we used the real dataset for training, then tested using a surrogate dataset). We restricted training or testing to frames in which mice were scored as actively engaged in social interaction (or matched frames during periods when the mouse was alone in its home cage). We also limited training/testing to frames with at least 3 active neurons.

We trained the output weights by performing 500 passes through the training data (each pass visited all of the training frames in a random order). On each training time step, we calculated *y*, the activity of the output unit, and then adjusted each output weight based on

$$\Delta w_{i}=\varepsilon y(1-y)(z-y)x_{i},$$

where z is the correct classification of the frame (0 for social behavior and 1 for home cage) and *ε*, the learning rate, was set to 0.05.

Following training, we examined the pattern of input connections and output weights. The distribution of output weights was roughly Gaussian and centered near 0. We identified the selection of prefrontal neurons most likely to be connected to hidden layer units with large positive or negative weights. Hidden layer units with large negative or positive output weights bias classification toward the social or home cage condition, respectively. Therefore, we refer to the 25 hidden units with the most negative or positive weights as “social” or “home cage” units respectively. We calculated the number of input connections between each prefrontal neuron and the 25 home cage units or 25 social units. We then defined “home cage” or “social” ensembles as the 20% of prefrontal neurons with the most input connections to home cage or social units, respectively. As described in the main text, we then analyzed properties of these 2 ensembles.

### Quantification of multineuron combinations

Estimating chance overlap between the activity of largely independent neurons requires accounting for 2 factors. First, neurons with higher activity are more likely to overlap by chance with other neurons. Second, overall network activity is dynamic over time, creating a tendency for otherwise independent neurons to be recruited at similar times. Thus, it is necessary to identify combinations which occur more often than expected based on (1) the activity levels of the constituent neurons; and (2) the fact that activity in a network is not constant over time. We can do this by quantifying the occurrence of combinations in datasets which have been shuffled to preserve (1) the overall level of activity in each neuron; and (2) the total level of activity in the network at each point in time.

Three-neuron combinations were quantified by identifying each combination present in frames in which 3 or more neurons were active. The number of frames each combination was active in real data was stored in an n-dimensional matrix. Surrogate datasets were then generated from event rasters by swapping the identity of neurons associated with detected events (periods of activity). As the timing of events themselves is unchanged, and only the identity of the participating neurons are exchanged, this preserves both the number of events per frame and the number of events that each neuron participates in. Therefore, the total number of combinations in each frame and over the course of the experiment (i.e., the sum of occurrences across all combinations) is also preserved. The total number of combination occurrences in which a given neuron participates would also tend to be preserved in these swap-shuffled surrogate datasets.

We then quantified how often each combination occurred in real versus swap-shuffled data. By comparing how often each combination occurred in real data versus in 1,000 swap-shuffled surrogate dataset, we were able to quantify how “enriched” each combination was compared to the level of occurrence expected by chance based on the activity levels of its constituent neurons (and the overall temporal pattern of network activity). We expressed enrichment as a percentile, calculated relative to swap-shuffled surrogate data, e.g., the 100th percentile indicates that a particular combination occurred more often in real data than in all 1,000 surrogate datasets. Further analysis was restricted to “enriched combinations,” i.e., combinations that occurred more often in real datasets than in 95% of surrogate datasets.

### Generation of synthetic datasets

All synthetic datasets consisted of 100 neurons and 6,000 frames with an overall activity A of 5%. Briefly, we randomly chose n active neurons in each frame f, where *n* = *A + sin*(*f*)*A*. Nonoverlapping patterns or assemblies each consisting of 8 neurons were inserted into this oscillating network reciprocally swapping activity from non-assembly neurons to assembly neurons in each frame in which the first neuron of the assembly/pattern was active. In frames in which less than 8 neurons were active, we randomly chose neurons from the assembly to be activated. To maintain equivalent levels of activity, reciprocal swaps were made by choosing another frame in which the assembly neuron receiving activity was active and reassigning activity in that second frame from the assembly neuron to the non-assembly neuron. To create datasets in which the activity of individual neurons was modulated between “State A” and “State B,” we transferred a proportion of active events from half the neurons to partner neurons so that half the neurons increased their activity and half the neurons activity decreased. For each pair of neurons, the proportion of transferred activity was a random number between 0 and the specified upper bounds of modulation which ranged between 5% and 50%. We trained our classifier on blocks consisting of 50% of each dataset and testing on the remainder. Each datapoint is the average of 10 iterations for each of 4 simulated datasets.

### SVM and logistic regression

For classification of synthetic datasets using logistic regression or support vector machines, we used the same training and testing blocks from the same networks as were tested using our neural network classifier. Models were trained using the command “fitclinear” in MATLAB. As before, each datapoint is the average of 10 iterations at each of 4 simulated datasets.

### Statistical analysis

Neurons and significant combinations from all animals and groups were pooled and counted as single units. Proportions were compared using chi-squared test. Activity levels were compared using paired *t* tests (2-sided), unless otherwise noted. Where applicable, error bars denote standard error of the mean. Values of the classifier performance (accuracy) were generated by averaging after rerunning the training/testing procedure at least 25 times. All datapoints showing classifier performance were averaged over at least 25 runs. When we specifically measured classifier performance on surrogate datasets, we averaged over 10 runs for each of 10 independently generated surrogate datasets (e.g., a total of 100 separate runs). For analyses of synthetic datasets, we generated 5 independent synthetic 2-state datasets for each level of activity modulation or assembly insertion. Each datapoint represents the results of training on the first dataset and testing on the latter 4 datasets (i.e., we used different datapoints for training versus testing), yielding 4 independent training–testing pairs; the accuracy of the classifier on each of these pairs was averaged over 10 runs of the classifier (e.g., a total of 40 separate runs).

Plots showing examples of classifier organization (rather than quantifying classifier performance) were derived from individual runs of the classifier (e.g., **Fig 3A–3E**, **S3 and S4 Figs**). **Fig 3F and 3G** shows changes in activity levels and correlations calculated based on ensembles derived from 1 run in each mouse, then averaged across all mice.

## Supporting information

S1 Fig Spatial decorrelation of neuronal signals. **(A)** Example image (top) and individual neuron GCaMP traces (bottom) from prefrontal cortex imaged with implanted endoscope. **(B)** The average GCaMP signal from an ROI, corresponding to 1 neuron, was corrected by subtracting the average GCaMP signal from the surrounding pixels, in order to spatially deconvolve signals from each ROI vs. the surrounding neuropil. Examples traces from a single neuron are shown. **(C)** The pairwise correlation matrix between signals from different neurons is shown (calculated from 550 seconds of activity from a single WT mouse), for the original GCaMP signals (top left), the surround signals (top right), the surround-subtracted signals (bottom left), and the surround-subtracted signals after low-pass filtering (bottom right). Data used to generate this figure can be found in the Supporting information Excel spreadsheet (S1 Data). ROI, region of interest; WT, wild-type.
(EPS)

S2 Fig Patterns of correlations and the accuracy of classifications that are based on correlated activity are both intact after SHARC shuffling. **Top:** As described in the main text, we created synthetic datasets comprising activity of 100 neurons during 2 states. The raster for “State B” was generated from the “State A” raster by inserting 1–5 assemblies composed of coactive neurons. Both the overall level activity in each neuron and total number of neurons active in each frame were unchanged between the “State A” and “State B” rasters. Left column: the first 200 frames of activity for “State A” and “State B,” followed by swap or SHARC-shuffled versions of the original “State B” raster. Middle column: matrices showing the pattern of inter-neuronal correlations for each raster. Right column: accuracy of a neural network trained to classify the original “State A” and “State B” rasters, tested using either the original “State B” raster or swap or SHARC-shuffled versions. Different colors = different numbers of inserted assemblies. Note that SHARC, but not swap shuffling, maintains both the pattern of correlations as well as the ability of the classifier to accurately decode “State A” vs. “State B.” **Bottom:** Similar to Top, but now we created the “State B” raster from the “State A” raster by randomly shifting a fraction of activity from neurons 1–50 to neurons 51–100. Thus, “State A” and “State B” differ in the activity levels of individual neurons. Different colors = different amounts of shifted activity. Note that in this case, the classifier functions independently of correlations and is not affected by swap shuffling the “State B” raster. SHARC, SHuffling Activity to Rearrange Correlations.
(EPS)

S3 Fig Neural networks classify states which differ in neuronal activity levels using ensembles of neurons that increase/decrease their activity in each state. **(A)** In this case, the State B raster was generated from the State A raster by shifting activity from neurons 1–50 to neurons 51–100. As a result, there is an increase (neurons 51:100) or decrease (neurons 1:50) in activity levels in State B compared to State A. Total neuronal activity in each frame is unchanged. **(B)** Matrix showing connections from input neurons to each of 1,000 hidden units. The hidden units have been ordered based on the output weights that are learned during classifier training. **(C)** After ordering the hidden units based on their output weight, we binned them into groups of 50. We calculated the average probability that each input neuron connects to hidden units in each bin. Neurons 51:100 (which have higher activity in State B) are likely to be connected to units with strong negative weights, whereas neurons 1:50 (which have lower activity in State B) are more likely to be connected to units given strong positive weights. **(D)** We defined negative and positive ensembles, composed of input neurons which tend to connected to hidden units in the first (most negative output weights) or last (most positive output weights) bin. (“State A” is classified as output = 1, whereas “State B” is classified as output = 0). **(E)** We calculated the activity of each ensemble (negative–left; positive–right) in each frame in either State A (red trace) or State B (blue trace). The negative ensemble (neurons which provide input to hidden units with negative weights) has higher activity in State B (left). Conversely, the positive ensemble has lower activity in State B. Note again that the red and blue traces depicted show the activity of a subset of neurons within an ensemble; total network activity in each frame is matched between State A and State B. **(F)** Cumulative distribution plot for activity of the positive and negative ensembles in State A (red trace) or State B (blue trace). The positive ensemble has higher activity in State B evidenced by rightward shift of the cumulative distribution function (right panel). The negative ensemble has lower activity in State B (left panel). CDF plots represent data from 4 simulations at the maximum level of activity modulation (up to 50% of activity shifted from neurons 1–50 to neurons 51–100). Data used to generate this figure can be found in the Supporting information Excel spreadsheet (S1 Data). CDF, cumulative distribution function.
(EPS)

S4 Fig Neural networks use diverse strategies to classify states which differ in patterns of coactivity but not in activity levels. **A**. We generated 2 states of activity. The “State A” raster was randomly generated (such that the total network activity oscillates over time). The “State B” raster was generated from the State A raster by “inserting” an assembly of coactive neurons (while keeping the total number of active neurons per frame and the total activity of each neuron fixed). We trained a neural network to classify frames in “State A” vs. those in “State B.” **(B, D)** For 2 example of neural networks trained to perform this classification, we plotted the matrix of connections from each input neuron to each hidden unit (left) or the average connection probability between each input neuron and a group of 50 hidden units (middle). Hidden units were sorted by the output weight learned during training, from low (most negative) to high (most positive). Finally, we identified the input neurons that were most likely to be connected to the group of hidden units with most negative weights (negative ensemble, “−”) or most positive weights (positive ensemble, “+”). In Example 1 (**B**), coactive assembly neurons are connected to hidden units with large negative weights, i.e., part of the negative ensemble. In Example 2 (**D**), coactive assembly neurons are connected to hidden units with strong positive weights, i.e., part of the positive ensemble. Because frames from “State A” were classified as output = 1 and “State B” frames were classified as output = 0, the negative ensemble biases the classifier toward “State B,” and the positive ensemble biases the classifier toward “State A.” **(C, E)** We calculated the activity of each ensemble (negative–left; positive–right) in each frame in either State A (red trace) or State B (blue trace) for Example 1 (B) or 2 (D). Note that in Example 1, the negative ensemble has increased activity in State B frames in which the assembly neurons are coactive (C, left plot, blue trace). By contrast, in Example 2, the positive ensemble is more active in these frames (E, right plot, blue trace). Thus, the coactive assembly neurons have opposing patterns of connectivity in these 2 examples, indicating that the corresponding neural networks are using 2 different strategies to classify State A vs. State B.
(EPS)

S5 Fig Behaviorally specific patterns of coactivity are formed by neurons that are active in both conditions, but coactive with different partners in each condition. **Left:** We identified combinations of 3 neurons that are specifically enriched during one behavioral condition (occurring more often during social interaction than in 95% of surrogate datasets and occurring less often during HC exploration than in 50% of surrogate datasets or vice versa). We then identified overlapping combinations occurring during the opposite behavioral condition in which a single neuron was “left out.” In other words, we identified combinations from the 2 conditions that overlapped in exactly 2 neurons. Based on these criteria, 12,408 3-neuron combinations were specifically enriched during social interaction, and 9,572 were specifically enriched during HC exploration. There were 55,696 instances in which a social and nonsocial 3-neuron combination overlapped in 2 out of 3 neurons. In 97.0% of these cases, the neuron which was part of a social 3-neuron combination (triplet) but left out of the overlapping HC triplet was part of a different 3-neuron combination that was enriched during HC exploration. Conversely, the neuron which was part of a nonsocial triplet but left out of the overlapping social 3-neuron combination was part of a different socially enriched 3-neuron combination in 99.1% of cases (S5 Fig, bottom right). Overall, an average of 71 enriched HC combinations contained the neuron missing from the social triplet, and 85 enriched social combinations contained the neuron missing from HC triplets. **Right, top:** Histogram showing the number of distinct 3 neuron HC combinations that contain the neuron which participates in a social combination but is “left out” during HC behavior. Overall, an average of 71 enriched HC combinations contained the neuron missing from the social triplet. **Right, bottom:** Histogram showing the number of distinct 3 neuron social combinations that contain the neuron which participates in a HC combination but is “left out” during social interaction. Overall, an average of 85 enriched social combinations contained the neuron missing from HC triplets. HC, home cage.
(EPS)

S1 Table Details for each mouse included in this study. The table shows the genotype, sex, number of imaged neurons, and peak classifier accuracy (performance when half the data was used for training and half for testing). **Fig 2** showed how classifier accuracy depends on the input connection probability; because their performance was not >50% for multiple input connection probabilities, WT mice 3 and 5 (marked with #) were not included in this illustrative plot. However, we did not exclude data from these mice in any analyses. WT mice 1–5 were WT littermates of *Shank3* KO mice. WT mice 6–10 (indicated by italics) were not littermates of *Shank3* KO mice and were therefore not included as WT controls for the analyses shown in **Fig 6**. KO, knockout; WT, wild-type.
(TIF)

S1 Data Data used to generate plots in figures. This Excel file contains multiple sheets, each of which contains the data that were used to generate plots in **Figs 1–6** (except **Fig 3C and 3E**) and **S1 and S3 Figs** (separated into different sheets for each figure panel).
(XLSB)

S2 Data Data used to generate plots in Fig 3C–3E. This Excel file contains a single sheet, containing the data that were used to generate plots in **Fig 3C–3E**. This is separate from S1 Data file due to file size.
(XLSB)

## Abbreviations

AP: anterior–posterior
DV: dorsoventral
IACUC: Institutional Animal Care and Use Committee
ICA: independent component analysis
KO: knockout
ML: mediolateral
mPFC: medial prefrontal cortex
NIH: National Institutes of Health
PCA: principal component analysis
ROI: region of interest
SHARC: SHuffling Activity to Rearrange Correlations
SNR: signal-to-noise ratio
SVM: support vector machine
UCSF: University of California, San Francisco
WT: wild-type

## References

[1] Cai DJ, Aharoni D, Shuman T, Shobe J, Biane J, Song W, et al. A shared neural ensemble links distinct contextual memories encoded close in time. Nature. 2016. 10.1038/nature17955 27251287 PMC5063500

[2] Liang B, Zhang L, Barbera G, Fang W, Zhang J, Chen X, et al. Distinct and Dynamic ON and OFF Neural Ensembles in the Prefrontal Cortex Code Social Exploration. Neuron [Internet]. 2018 11 7 [cited 2019 Mar 30];100(3):700–14.e9. Available from: https://www.sciencedirect.com/science/article/pii/S0896627318307724?via%3Dihub 10.1016/j.neuron.2018.08.043 30269987 PMC6224317

[3] deCharms RC, Merzenich MM. Primary cortical representation of sounds by the coordination of action-potential timing. Nature [Internet]. 1996 6 13 [cited 2019 Aug 25];381(6583):610–3. Available from: http://www.ncbi.nlm.nih.gov/pubmed/8637597 10.1038/381610a0 8637597

[4] Hebb DO. The organization of behavior: A neuropsychological theory. New York: Wiley; 1949.

[5] Buzsáki G. Neural Syntax: Cell Assemblies, Synapsembles, and Readers. Neuron [Internet]. 2010 11 4 [cited 2019 Mar 30];68(3):362–85. Available from: http://www.ncbi.nlm.nih.gov/pubmed/21040841 10.1016/j.neuron.2010.09.023 21040841 PMC3005627

[6] De La Rocha J, Doiron B, Shea-Brown E, Josić K, Reyes A, Josi K, et al. Correlation between neural spike trains increases with firing rate. Nature. 2007. 10.1038/nature06028 17700699

[7] Ruff DA, Cohen MR. Global cognitive factors modulate correlated response variability between V4 neurons. J Neurosci [Internet]. 2014 12 3 [cited 2020 Oct 31];34(49):16408–16. Available from: https://www.jneurosci.org/content/34/49/16408 10.1523/JNEUROSCI.2750-14.2014 25471578 PMC4252550

[8] Averbeck BB, Latham PE, Pouget A. Neural correlations, population coding and computation. [cited 2020 Aug 2]. Available from: www.nature.com/reviews/neuro 10.1038/nrn1888 16760916

[9] Abbott LF, Dayan P. The effect of correlated variability on the accuracy of population code. 10.1162/089976699300016827 9950724

[10] Cohen MR, Maunsell JHR. Attention improves performance primarily by reducing interneuronal correlations. Nat Neurosci [Internet]. 2009 12 15 [cited 2019 Mar 18];12(12):1594–600. Available from: http://www.nature.com/articles/nn.2439 10.1038/nn.2439 19915566 PMC2820564

[11] Vaadia E, Haalman I, Abeles M, Bergman H. Dynamics of neuronal interactions in monkey cortex in relation to behavioural events. Nature [Internet]. 1995 2 9 [cited 2020 Jul 19];373(6514):515–8. Available from: https://www.nature.com/articles/373515a0 10.1038/373515a0 7845462

[12] Cheng S, Frank LM. New Experiences Enhance Coordinated Neural Activity in the Hippocampus. Neuron [Internet]. 2008 1 24 [cited 2021 Feb 4];57(2):303–13. Available from: https://pubmed.ncbi.nlm.nih.gov/18215626/ 10.1016/j.neuron.2007.11.035 18215626 PMC2244590

[13] Averbeck BB, Lee D. Effects of noise correlations on information encoding and decoding. J Neurophysiol [Internet]. 2006 6 [cited 2020 Aug 13];95(6):3633–44. Available from: www.jn.org 10.1152/jn.00919.2005 16554512

[14] Ahmed MS, Priestley JB, Castro A, Stefanini F, Solis Canales AS, Balough EM, et al. Hippocampal Network Reorganization Underlies the Formation of a Temporal Association Memory. Neuron. 2020 7 22;107(2):283–291.e6. 10.1016/j.neuron.2020.04.013 32392472 PMC7643350

[15] Stefanini F, Kushnir L, Jimenez JC, Jennings JH, Woods NI, Stuber GD, et al. A Distributed Neural Code in the Dentate Gyrus and in CA1. Neuron [Internet]. 2020 [cited 2020 Aug 2]. Available from: https://pubmed.ncbi.nlm.nih.gov/32521223/10.1016/j.neuron.2020.05.022 PMC7442694 32521223

[16] Siniscalchi MJ, Wang H, Kwan AC. Enhanced Population Coding for Rewarded Choices in the Medial Frontal Cortex of the Mouse. Cereb Cortex [Internet]. 2019 9 13 [cited 2020 Nov 3];29(10):4090–106. Available from: https://pubmed.ncbi.nlm.nih.gov/30615132/ 10.1093/cercor/bhy292 30615132 PMC6735259

[17] Yizhar O, Fenno LE, Prigge M, Schneider F, Davidson TJ, O’Shea DJ, et al. Neocortical excitation/inhibition balance in information processing and social dysfunction. Nature. 2011. 10.1038/nature10360 21796121 PMC4155501

[18] Selimbeyoglu A, Kim CK, Inoue M, Lee SY, Hong ASO, Kauvar I, et al. Modulation of prefrontal cortex excitation/inhibition balance rescues social behavior in CNTNAP2-deficient mice. Sci Transl Med [Internet]. 2017 8 2 [cited 2019 Aug 23];9(401):eaah6733. Available from: http://www.ncbi.nlm.nih.gov/pubmed/28768803 10.1126/scitranslmed.aah6733 28768803 PMC5723386

[19] Brumback AC, Ellwood IT, Kjaerby C, Iafrati J, Robinson S, Lee AT, et al. Identifying specific prefrontal neurons that contribute to autism-associated abnormalities in physiology and social behavior. Mol Psychiatry [Internet]. 2017 11 7 [cited 2018 Feb 12]. Available from: http://www.ncbi.nlm.nih.gov/pubmed/29112191 10.1038/mp.2017.213 29112191 PMC6594833

[20] Murugan M, Jang HJ, Park M, Miller EM, Cox J, Taliaferro JP, et al. Combined Social and Spatial Coding in a Descending Projection from the Prefrontal Cortex. Cell [Internet]. 2017 12 [cited 2019 May 20];171(7):1663–1677.e16. Available from: https://linkinghub.elsevier.com/retrieve/pii/S0092867417313119 10.1016/j.cell.2017.11.002 29224779 PMC5889923

[21] Levy DR, Tamir T, Kaufman M, Parabucki A, Weissbrod A, Schneidman E, et al. Dynamics of social representation in the mouse prefrontal cortex. Nat Neurosci [Internet]. 2019 11 25 [cited 2020 Jan 13]. Available from: http://www.ncbi.nlm.nih.gov/pubmed/31768051 10.1038/s41593-019-0531-z 31768051

[22] Mukamel EA, Nimmerjahn A, Schnitzer MJ. Automated analysis of cellular signals from large-scale calcium imaging data. Neuron [Internet]. 2009 9 24 [cited 2017 Jun 6];63(6):747–60. Available from: http://linkinghub.elsevier.com/retrieve/pii/S0896627309006199 10.1016/j.neuron.2009.08.009 19778505 PMC3282191

[23] Luongo FJ, Horn ME, Sohal VS. Putative microcircuit-level substrates for attention are disrupted in mouse models of autism. Biol Psychiatry. 2016. 10.1016/j.biopsych.2015.04.014 26022075 PMC4624609

[24] Luongo FJ, Zimmerman CA, Horn ME, Sohal VS. Correlations between prefrontal neurons form a small-world network that optimizes the generation of multineuron sequences of activity. J Neurophysiol [Internet]. 2016 5 1 [cited 2018 Feb 6];115(5):2359–75. Available from: http://www.ncbi.nlm.nih.gov/pubmed/26888108 10.1152/jn.01043.2015 26888108 PMC5394653

[25] Chen Q, Deister CA, Gao X, Guo B, Lynn-Jones T, Chen N, et al. Dysfunction of cortical GABAergic neurons leads to sensory hyper-reactivity in a Shank3 mouse model of ASD. Nat Neurosci [Internet]. 2020 3 2 [cited 2020 Mar 9];1–13. Available from: http://www.ncbi.nlm.nih.gov/pubmed/32123378 10.1038/s41593-019-0563-4 32123378 PMC7131894

[26] Peça J, Feliciano C, Ting JT, Wang W, Wells MF, Venkatraman TN, et al. Shank3 mutant mice display autistic-like behaviours and striatal dysfunction HHS Public Access. Nature [Internet]. 2011;472(7344):437–42. Available from: http://www.nature.com/authors/editorial_policies/license.html#terms 10.1038/nature09965 21423165 PMC3090611

[27] Duffney LJ, Zhong P, Wei J, Matas E, Cheng J, Qin L, et al. Autism-like Deficits in Shank3-Deficient Mice Are Rescued by Targeting Actin Regulators. Cell Rep. 2015. 10.1016/j.celrep.2015.04.064 26027926 PMC4464902

[28] Corder G, Ahanonu B, Grewe BF, Wang D, Schnitzer MJ, Scherrer G. An amygdalar neural ensemble that encodes the unpleasantness of pain. Science. 2019 1 18;363(6424):276–81. 10.1126/science.aap8586 30655440 PMC6450685

[29] Gründemann J, Bitterman Y, Lu T, Krabbe S, Grewe BF, Schnitzer MJ, et al. Amygdala ensembles encode behavioral states. Science [Internet]. 2019 4 19 [cited 2019 Jul 8];364(6437):eaav8736. Available from: http://www.ncbi.nlm.nih.gov/pubmed/31000636 10.1126/science.aav8736 31000636

[30] Ghandour K, Ohkawa N, Fung CCA, Asai H, Saitoh Y, Takekawa T, et al. Orchestrated ensemble activities constitute a hippocampal memory engram. Nat Commun [Internet]. 2019 12 14 [cited 2019 Jul 8];10(1):2637. Available from: http://www.nature.com/articles/s41467-019-10683-2 10.1038/s41467-019-10683-2 31201332 PMC6570652

[31] Sakurai Y. How do cell assemblies encode information in the brain? Neurosci Biobehav Rev. 1999. 10.1016/s0149-7634(99)00017-2 10541056

[32] Sakurai Y, Osako Y, Tanisumi Y, Ishihara E, Hirokawa J, Manabe H. Multiple approaches to the investigation of cell assembly in memory research—present and future. Vol. 12, Frontiers in Systems Neuroscience. Frontiers Media S.A.; 2018. 10.3389/fnsys.2018.00021 PMC5980992 29887797

[33] Pinto L, Goard MJ, Estandian D, Xu M, Kwan AC, Lee S-H, et al. Fast modulation of visual perception by basal forebrain cholinergic neurons. 2013;16(12). 10.1038/nn.3552 PMC4201942 24162654

[34] Dadarlat MC, Stryker MP. Locomotion enhances neural encoding of visual stimuli in mouse V1. J Neurosci [Internet]. 2017 4 5 [cited 2020 Jul 29];37(14):3764–75. Available from: https://www.jneurosci.org/content/37/14/3764 10.1523/JNEUROSCI.2728-16.2017 28264980 PMC5394894

[35] Abeles M, Bergman H, Gat I, Meilijson I, Seidemann E, Tishby N, et al. Cortical activity flips among quasi-stationary states. Proc Natl Acad Sci U S A. 1995 9 12;92(19):8616–20. 10.1073/pnas.92.19.8616 7567985 PMC41017

[36] Schneidman E, Berry MJ, Segev R, Bialek W. Weak pairwise correlations imply strongly correlated network states in a neural population. Nature [Internet]. 2006 4 20 [cited 2020 Aug 5];440(7087):1007–12. Available from: https://pubmed.ncbi.nlm.nih.gov/16625187/ 10.1038/nature04701 16625187 PMC1785327

[37] Jiang X, Shen S, Cadwell CR, Berens P, Sinz F, Ecker AS, et al. Principles of connectivity among morphologically defined cell types in adult neocortex. Science. 2015 11 27;350(6264). 10.1126/science.aac9462 26612957 PMC4809866

[38] Alex M. Thomson, David C. West, Yun Wang APB. Synaptic Connections and Small Circuits Involving Excitatory and Inhibitory Neurons in Layers 2–5 of Adult Rat and Cat Neocortex: Triple Intracellular Recordings and Biocytin Labelling In Vitro. Cereb Cortex [Internet]. 2002 [cited 2020 Jul 29];12(9):936–53. Available from: https://academic.oup.com/cercor/article/12/9/936/383201 10.1093/cercor/12.9.936 12183393

[39] Ko H, Hofer SB, Pichler B, Buchanan KA, Sjöström PJ, Mrsic-Flogel TD. Functional specificity of local synaptic connections in neocortical networks. Nature [Internet]. 2011 5 10 [cited 2019 Aug 25];473(7345):87–91. Available from: http://www.ncbi.nlm.nih.gov/pubmed/21478872 10.1038/nature09880 21478872 PMC3089591

[40] Litwin-Kumar A, Doiron B. Slow dynamics and high variability in balanced cortical networks with clustered connections. Nat Neurosci [Internet]. 2012 11 [cited 2019 Aug 23];15(11):1498–505. Available from: http://www.ncbi.nlm.nih.gov/pubmed/23001062 10.1038/nn.3220 23001062 PMC4106684

[41] Hamm JP, Peterka DS, Gogos JA, Yuste R. Altered Cortical Ensembles in Mouse Models of Schizophrenia. Neuron [Internet]. 2017 4 5 [cited 2017 Jun 6];94(1):153–167.e8. Available from: http://www.ncbi.nlm.nih.gov/pubmed/28384469 10.1016/j.neuron.2017.03.019 28384469 PMC5394986

[42] Nelson SB, Valakh V. Excitatory/Inhibitory Balance and Circuit Homeostasis in Autism Spectrum Disorders. Neuron [Internet]. 2015 8 19 [cited 2019 May 19];87(4):684–98. Available from: http://www.ncbi.nlm.nih.gov/pubmed/26291155 10.1016/j.neuron.2015.07.033 26291155 PMC4567857

[43] Rubenstein JLR, Merzenich MM. Model of autism: increased ratio of excitation/inhibition in key neural systems. Genes, Brain Behav. 2003. 10.1034/j.1601-183x.2003.00037.x 14606691 PMC6748642

[44] Chen T-W, Wardill TJ, Sun Y, Pulver SR, Renninger SL, Baohan A, et al. Ultrasensitive fluorescent proteins for imaging neuronal activity. Nature [Internet]. 2013 7 18 [cited 2019 Apr 17];499(7458):295–300. Available from: http://www.ncbi.nlm.nih.gov/pubmed/23868258 10.1038/nature12354 23868258 PMC3777791

