# Supplementary figures and images for: Dynamic patterns of correlated activity in the prefrontal cortex encode information about social behavior
Source: PLoS Biol. 2021 May 3;19(5):e3001235. doi: 10.1371/journal.pbio.3001235 (PMC8118626; doi:10.1371/journal.pbio.3001235)

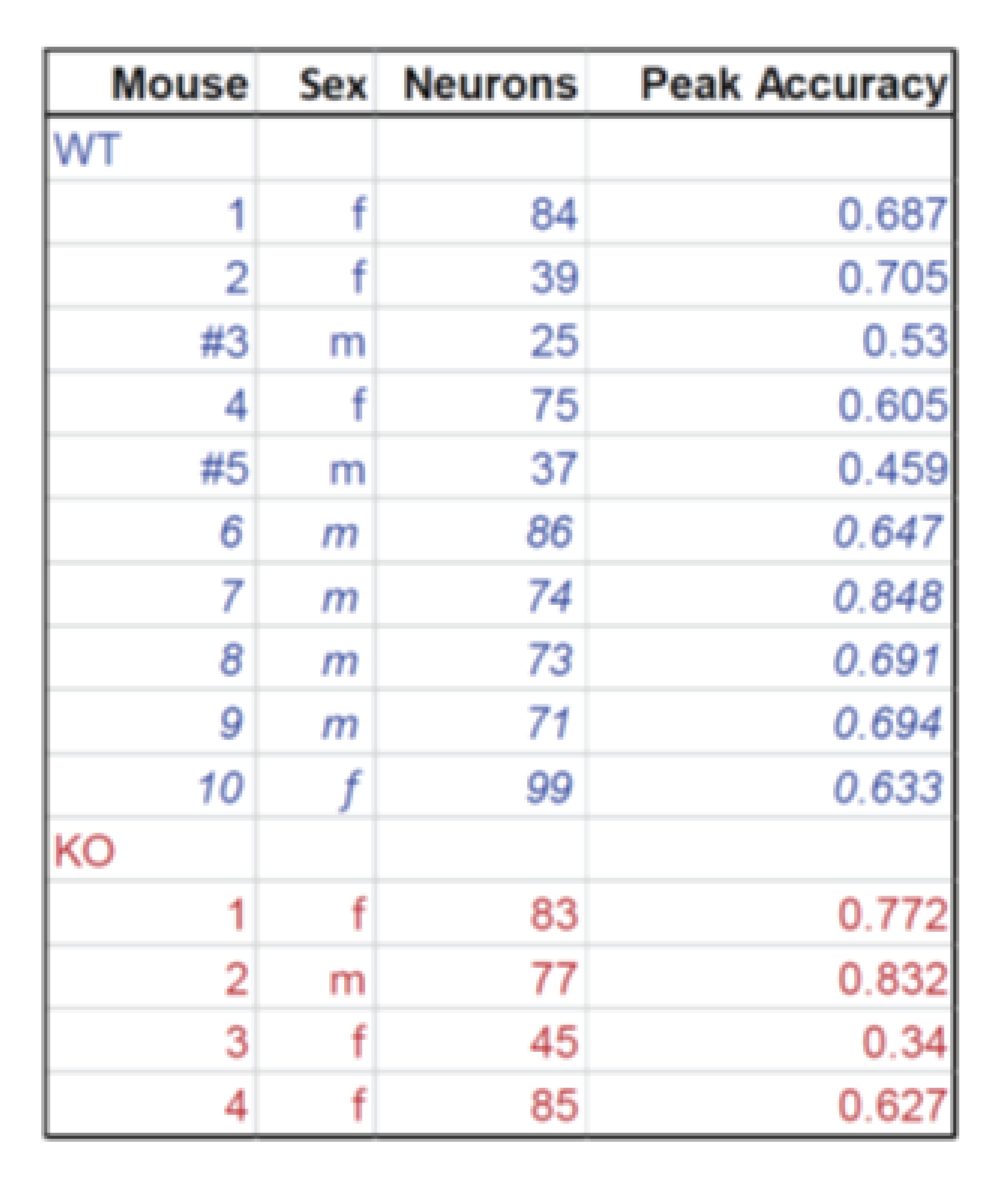

Supplement: S1 Table — The table shows the genotype, sex, number of imaged neurons, and peak classifier accuracy (performance when half the data was used for training and half for testing). Fig 2 showed how classifier accuracy depends on the input connection probability; because their performance was not >50% for multiple input connection probabilities, WT mice 3 and 5 (marked with #) were not included in this illustrative plot. However, we did not exclude data from these mice in any analyses. WT mice 1–5 were WT littermates of Shank3 KO mice. WT mice 6–10 (indicated by italics) were not littermates of Shank3 KO mice and were therefore not included as WT controls for the analyses shown in Fig 6. KO, knockout; WT, wild-type. (TIF) [file pbio.3001235.s006.tif]
